# Persistent expression of *Cotesia plutellae* bracovirus genes in parasitized host, *Plutella xylostella*

**DOI:** 10.1371/journal.pone.0200663

**Published:** 2018-07-16

**Authors:** Yonggyun Kim, Sunil Kumar

**Affiliations:** Department of Plant Medicals, Andong National University, Andong, Korea; Institute of Plant Physiology and Ecology Shanghai Institutes for Biological Sciences, CHINA

## Abstract

Cotesia plutellae (= vestalis) bracovirus (CpBV) is symbiotic to an endoparasitoid wasp, *C*. *plutellae*, and plays crucial roles in parasitism against the diamondback moth, *Plutella xylostella*. CpBV virion genome consists of 35 circular DNAs encoding 157 putative open reading frames (ORFs). This study re-annotated 157 ORFs with update genome database and analyzed their gene expressions at early and late parasitic stages. Re-annotation has established 15 different viral gene families, to which 83 ORFs are assigned with remaining 74 hypothetical genes. Among 157 ORFs, 147 genes were expressed at early or late parasitic stages, among which 141 genes were expressed in both parasitic stages, indicating persistent nature of gene expression. Relative frequencies of different viral circles present in the ovarian lumen did not explain the expression variation of the viral ORFs. Furthermore, expression level of each viral gene was varied during parasitism along with host development. Highly up-regulated CpBV genes at early parasitic stage included BEN (BANP, E_5_R and NAC_1_), ELP (EP1-like protein), IkB (inhibitor kB), P494 (protein 494 kDa) family genes, while those at late stage were mostly hypothetical genes. Along with the viral gene expression, 362 host genes exhibited more than two fold changes in expression levels at early parasitic stage compared to nonparasitized host. At late stage, more number (1,858) of host genes was regulated. These results suggest that persistent expression of most CpBV genes may be necessary to regulate host physiological processes during *C*. *plutellae* parasitism.

## Introduction

Polydnaviruses (PDVs) are symbiotic to some endoparasitoid wasps and classified into bracoviruses (BVs) and ichnoviruses (IVs) depending on hymenopteran host families [1]. PDV genome is located on the host wasp chromosome(s) as a proviral form [2]. Both BV and IV have been hypothesized to be originated from different ancestral insect viruses [3]. With respect to the origin of BVs, an ancestral form of nudiviruses is likely to have infected a common ancestor wasp and integrated its genome into the wasp chromosome(s) at about 100 million years ago [4]. During domestication of the ancestral nudivirus, an essential gene set for BV replication has been retained, but not incorporated into viral particles [5]. In contrast, the viral particles may have evolved to harbor parasitism-associated genes derived from virulent host genes probably via horizontal gene transfer [6]. Thus BV genome consists of two parts: nudiviral and proviral genes [7]. However, an ancestral viral identity is still unknown in IVs, though their proviral genes have been identified in several species [8,9].

BVs are symbiotic to about 17,500 described wasp species of a monophyletic wasp group called a microgastroid complex containing 7 subfamilies (Cheloninae, Dirrhopinae, Mendesellinae, Khoikhoiinae, Cardiochilinae, Miracinae, and Microgastrinae) [10]. This large number of BV hosts is believed to have originated from an association of nudiviral incorporation into a wasp species and then diversified [11]. This species diversification requires successful parasitism against diverse hosts. As seen in an example of *Cotesia sesamiae* against susceptible and resistant hosts, BV proviral genes have been positively selected to protect wasp hosts probably from immune attack of parasitized hosts [12]. In fact, a BV proviral genome consists of multiple PDV gene families presumably parasitizing multiple hosts to defend various immune defenses and alter host development to facilitate successful parasitism of host wasps [3,6].

Cotesia plutellae (= vestalis) bracovirus (CpBV) is symbiotic to an endoparasitoid wasp, *C*. *plutellae*, parasitizing young larvae of the diamondback moth, *P*. *xylostella* [13]. Parasitized host exhibits significant immunosuppression and developmental retardation [14,15]. CpBV proviral genome contains 157 putative open reading frames (ORFs) [16]. These ORFs are annotated into different PDV gene families with remaining hypothetical genes. Some ORFs have been assessed in gene expression in parasitized host and analyzed in physiological functions [17–20]. However, comprehensive transcriptome analysis of these CpBV ORFs has not been performed. Furthermore, recent accumulation of genome information raised a reannotation issue on CpBV proviral genome. Recent functional analyses of some CpBV genes have been also validated to form novel gene families in CpBV genome. Ali and Kim [21] proposed a BEN (BANP, E5R, and NAC1) family in CpBV and showed that 11 members of BEN family are expressed in parasitized larvae. Subsequently, these BEN family members were shown to be crucial in suppressing cellular and humoral immune responses of parasitized host [22]. Four p94-like baculoviral genes (early expressed p94s: E94Ks) were found in the CpBV genome and their physiological functions were assessed in suppressing immune and development of parasitized host [23]. This study re-annotated CpBV proviral genes and assessed all the 157 ORFs through genome-wide transcriptome analysis in the parasitized host. In addition, gene expression of the host insect during parasitism was monitored by RNA-Seq analysis to signify an effect of the viral gene expression on physiological changes of the parasitized host.

## Materials and methods

### 2.1. Insects and parasitization

*P*. *xylostella* larvae were reared under 25 ± 1°C and a 16:8 h (L:D) photoperiod with cabbage leaves. Adults were fed with 10% sucrose solution. About 200 late first instar larvae (3 days after hatch at 25°C) were parasitized by about 100 *C*. *plutellae* adults for 24 h. Under this condition, parasitization rate was recorded over 95% [13]. The parasitized larvae were reared on cabbage leaves at the rearing environment. After adult emergence, wasps were allowed to mate for 24 h and then again used for parasitization.

### 2.2. RNA extraction for RNA-Seq

Based on the time of parasitization, parasitized (P) larvae of *P*. *xylostella* lived for 8 days at 25°C and then died just before pupation. In contrast, nonparasitized (NP) larvae at the corresponding stage of parasitized larvae lived for 6 days and then pupated. Under the same developmental conditions, test insects were selected at P1 (one day after parasitization) and P7 (seven days after parasitization) stages. P1 was at second instar larvae while P7 was at late fourth instar. For comparison, test larvae in NP groups were selected at NP1 (corresponding to P1) and NP5 (corresponding to P7). Total RNA was extracted using Trizol reagent (Invitrogen, Carlsbad, CA, USA) with about 100 young larvae (P1 or NP1) or about 20 old larvae (P7 or NP5) due to body size difference. Extracted RNA was resuspended in 40 μL diethyl pyrocarbonate-treated water. RNA integrity for subsequent RNA-Seq was analyzed using Bioanalyzer 2100 (Agilent Technologies, Santa Clara, CA, USA) in Macrogen Inc. (Seoul, Korea).

### 2.3. RNA-Seq and data processing

From total RNA extracted as above, cDNA library was constructed with Truseq RNA Library Preparation Kit (Illumina Inc., San Diego, CA, USA) and sequenced in a paired-end mode by Illumina HiSeq 2000 in an 101 bp read length. Raw reads were trimmed by Trimmomatic 0.32 program (http://www.usadellab.org/cms/?page=trimmomatic) under a criterion of 230 (phred score base quality 30% or more) and then mapped on CpBV genome [16] or DBM genome (http://iae.fafu.edu.cn/DBM/index.php) using TopHat program (version 2.0.13) (http://ccb.jhu.edu/software/tophat/index.shtml) and Bowtie (version 2 2.2.3). From these mappings, NP1, NP5, P1 and P7 samples recorded 58%, 55%, 54% and 26% mapping ratios (S1 Table). The mapped reads after trimming were then assembled with Cufflinks (version 2.2.1) (http://cole-trapnell-lab.github.io/cufflinks/) and calculated into FPKM (fragment per kilobase of transcript per million mapped reads). For DEG (differential expression gene) analysis, 12,945 transcripts were used by deleting 5,128 transcripts from a total of 18,073 transcripts because at least one of samples contained FPKM values of 0. DEG used a criterion of at least two fold changes in FPKM values.

### 2.4. Re-annotation of CpBV ORFs

Based on earlier annotation [16], 157 ORFs were manually blasted to current database (August 20, 2016) of NCBI GenBank using BlastX program. To validate gene families, CpBV genes were compared with other PDV genes, which had been annotated into the corresponding gene families, by phylogenetic clustering analysis using MEGA6 [24] and ClustalW program of DNASTAR (Version 5.01). Phylogenetic tree was constructed with Neighbor-joining method and bootstrap values at branches were obtained with 500 repetitions.

### 2.5. Validation by RT-PCR and RT-qPCR

Total RNA was extracted as described above and cDNA was synthesized by adding 1 μg RNA into Maxime RT PreMix (iNtRON Biotechnology, Seoul, Korea) containing reverse transcriptase and oligo dT primer. The reaction mixture was incubated at 42°C for 90 min and then subjected to an inactivation step at 95°C for 5 min. PCR was conducted using DNA Taq polymerase (GeneAll, Seoul, Korea) under conditions: 94°C for 5 min followed by 35 cycles of 94°C for 1 min, 55°C for 30 s, and 72°C for 30–45 s depending on amplicon length with a final extension at 72°C for 7 min. Each RT-PCR reaction (25 μL) consisted of template cDNA and 10 pmol for each of forward and reverse primers (S2 Table). For an endogenous control a ribosomal protein, *RL32*, gene was used with the RNA extract as a template to confirm the absence of DNA contamination. All gene specific primer sequences are described in S2 Table.

For RT-qPCR, cDNA quantity was estimated using GeneQuant spectrophotometer (Model No. 80211504, Amersham Biosciences, Science Park, Singapore) and diluted to 50 ng/μL. A PCR in 20 μL reaction volume consisted of 2× SYBR^®^ Green Realtime PCRMasterMix (Code QPK-201, TOYOBO, Osaka, Japan), 5 μM of gene-specific forward and reverse primers (S1 Table), in which 50 ng of cDNA was used as template. PCR was performed at 95°C for 10 min for an initial denaturation and followed by 40 cycles at 98°C for 15 s, 54°C for 30 s, and 72°C for 45 s with a final extension at 72°C for 7 min. Melting curves were assessed to confirm the unique PCR products. The ΔΔCT method [25] was used for the calculation of the relative mRNA expression levels. *RL32* gene expression was used for normalization.

## Results

### 3.1. Re-annotation of CpBV encapsidated genome

This study was intended to analyze the expressions of all 157 CpBV genes in parasitized host. Before this expression analysis, we needed to re-annotate CpBV genes with current GenBank database and recent functional studies (see Discussion) related with their genes. This re-annotation shows that more than 50% of ORFs (83 genes) are assigned to 15 gene families characterized by viral and eukaryotic conserved sequences, while 74 ORFs are still hypothetical in functional annotation (Table 1). Compared to previous annotation [16], 16 ORFs that were classified into hypothetical (HP) genes are now assigned to new gene families of E94K, P494, and P325. RNaseT2 family is now combined with BEN family because several BEN family genes possess RNaseT2 domain.

**Table 1:** A revised annotation of 157 CpBV ORFs from 35 circles (C1-C35). Their sequences are obtained from GenBank with accession numbers of HQ009524-HQ009558.

Gene families	Revision	Chen et al. (2011)^1^
Hypothetical gene (HP)	74	90
Protein tyrosine phosphatase (PTP)	33	33
BEN	12	11^2^
Inhibitor kappa B (IkB)	7	6
EP1-like protein (ELP)	7	6
Serine-rich protein (SRP)	6	2
E94K	5	0
Cysteine-rich protein (CRP)	3	2
C type lectin (CTL)	2	2
P494	2	0
CrV1	1	1
Cystatin (CST)	1	1
Viral histone H4 (vH4)	1	1
Duffy-binding (DUFB)	1	1
DNA helicase (dHEL)	1	1
P325	1	0

^1^ Chen et al. (2011) denotes a previous annotation of CpBV genome.

^2^ A family of RNaseT2 in Chen et al. (2011) is now combined with BEN family.

**Fig 1:**
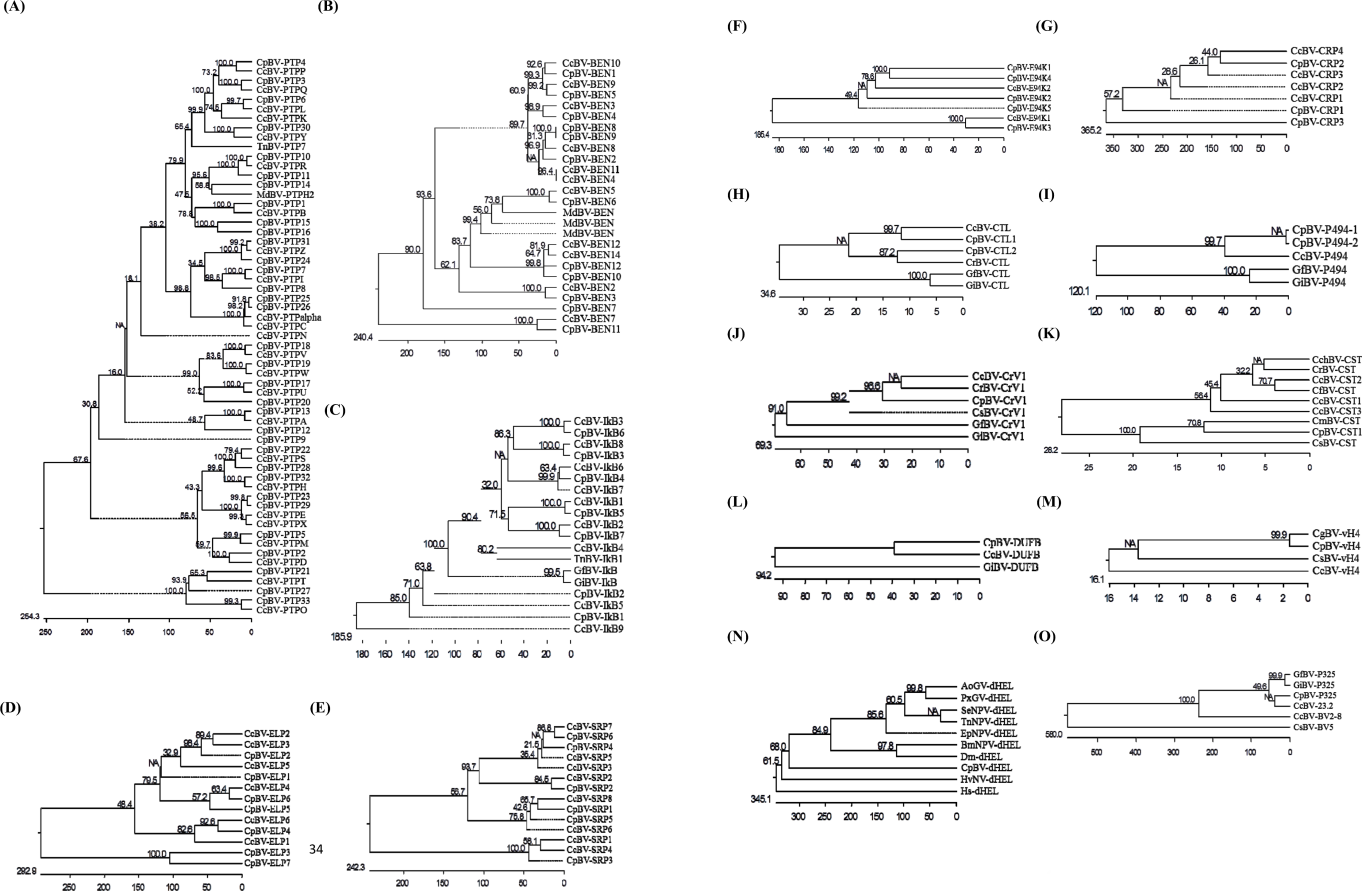
**Phylogenetic analyses of 15 CpBV gene families:** (A) protein tyrosine phosphatase (PTP) (B) BANP, E5R and NAC1 (BEN) (C) Inhibitor kB (IkB) (D) EP1-like protein (ELP) (E) Serine-rich protein (SRP) (F) E94K (G) Cysteine-rich protein (CRP) (H) C type lectin (CTL) (I) P494 (J) CrV1 (K) Cystatin-like (CST) (L) Duffy-binding (DUFB) (M) viral histone H4 (vH4) (N) DNA helicase (dHEL), and (O) P325. Amino acid sequences of these genes were retrieved from GenBank with accession numbers listed in S3 Table. Amino acid sequences were aligned with MEGA6 [24]. Bootstrap values on branches were obtained with 500 repetitions.

All CpBV gene families established in this study are supported by shared sequences (S1–S15 Figs) and clusterings with other PDV genes (Fig 1). PTP gene family consists of 33 members and exhibits co-clustering with other viral PTPs (Fig 1A). On the basis of conserved PTP residues, 23 members are presumed to be catalytically active, but 12 members have mutated residues from Cys in catalytic site to Gly or Ser. *CpBV-PTP3*, *-PTP4*, *-PTP6*, and *-PTP30* co-clustered with *TnBV-PTP17*, which was known to inhibit metamorphosis [26]. *CpBV-PTP14* clustered with *MdBV-PTPH2*, which was known to inhibit immune response [27]. In a similar phylogenetic analysis, other 13 gene families also exhibited homologies with the corresponding orthologs of CcBV, TnBV or MdBV (1B-O).

All HP genes were aligned and compared by a phylogenetic analysis (S16 Fig). HP genes can be classified into several subgroups. However, few branches except the shaded boxes are statistically supported in the phylogenetic analysis.

### 3.2. Most CpBV genes are expressed in parasitized host

**Fig 2:**
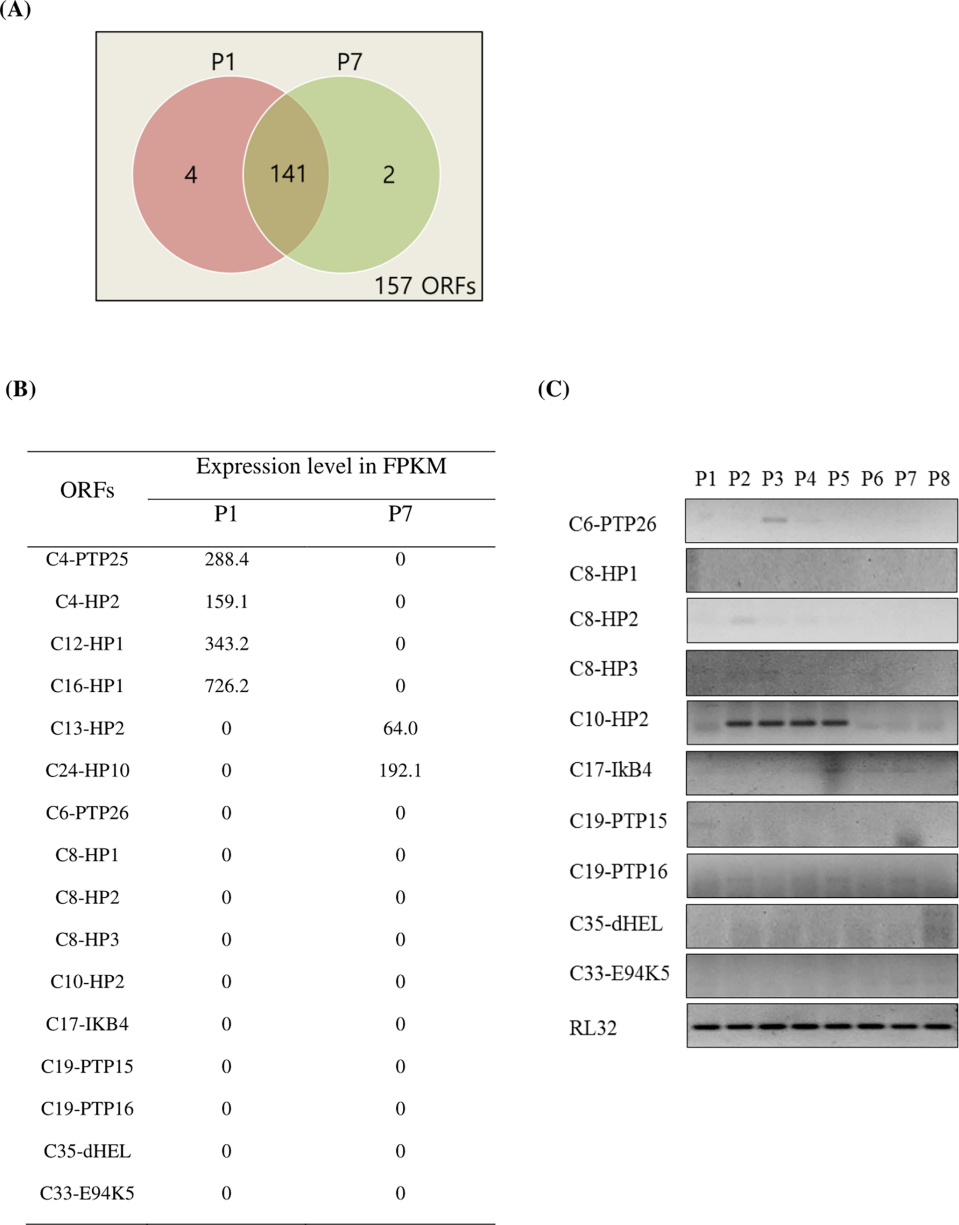
Expression analysis of 157 ORFs of CpBV in *P*. *xylostella* parasitized by *C*. *plutellae* using RNA-Seq by Illumina HiSeq. Two parasitic stages were assessed at early (one day after parasitization: P1) and late (7 days after parasitization: P7) stages. (A) Venn diagram showing the number of CpBV genes expressed in both parasitic stages. 141 ORFs are commonly expressed in both stages whereas 6 genes are expressed at P1 or P7. (B) List of CpBV genes not expressed at either or both stages whose expression level was shown in FPKM. (C) RT-PCR analysis of 10 CpBV unexpressed genes at both parasitic stages. A ribosomal protein, *RL32*, gene with its gene-specific primers (S1 Table) was used as a constitutively expressed reference gene.

After reannotation of CpBV ORFs, we tested whether all 157 ORFs would be expressed in the parasitized host by using total transcriptome analysis using RNA-Seq (Fig 2). Parasitized larvae at early (1 day old after parasitization: P1) and late (7 days old after parasitization: P7) parasitic stages were assessed. Among these 157 ORFs, 147 ORFs were expressed in two parasitic stages, in which 141 ORFs were expressed in both developmental stages (Fig 2A). Six CpBV genes were expressed at either early (P1) or late (P7) parasitic stages (Fig 2B). Some of the unexpressed 10 CpBV genes at both stages were expressed in other stages (Fig 2C), in which *C6-PTP26* at P3, *C8-HP2* at P2, *C10-HP2* was expressed at P2, P3, P4, P5, *C17-IkB* was expressed only at P5, and *C19-PTP16* was expressed at P2, P5, P7, whereas *C8-HP1*, *C8-HP3*, *C19-PTP15*, *C33-E94K5*, and *C35-dHEL* were not expressed at all.

**Fig 3:**
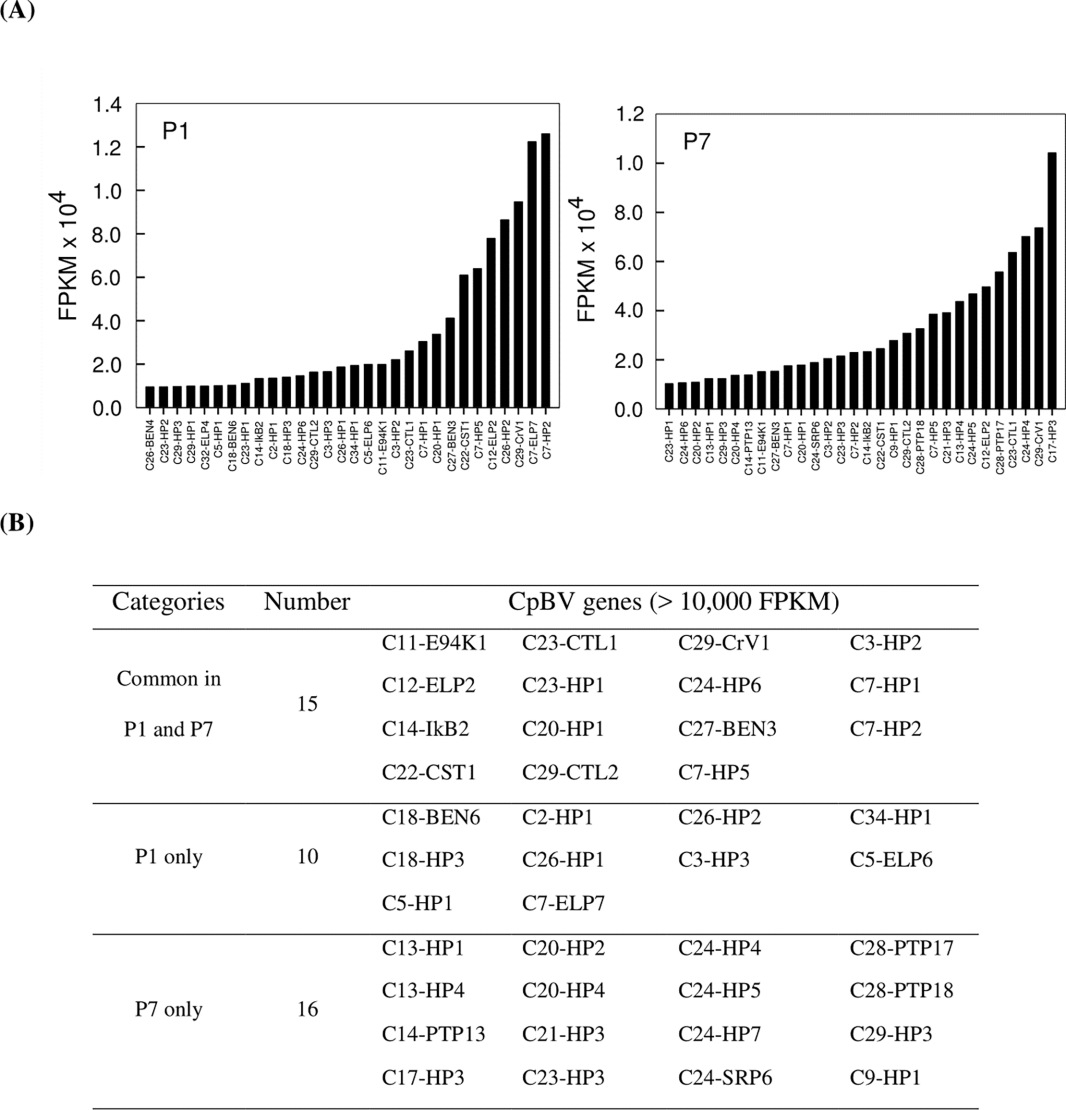
Highly expressed CpBV genes in *P*. *xylostella* parasitized by *C*. *plutellae*. Expression levels were calculated by FPKM based on read numbers obtained from Illumina HiSeq. (A) Highly expressed CpBV genes at early (one day after parasitization: P1) and late (7 days after parasitization: P7) parasitic stages. (B) Classification of highly expressed genes more than 10,000 scores in FPKM values.

However, highly expressed CpBV ORFs were different between P1 and P7 parasitic stages (Fig 3A). CpBV genes exhibiting 10,000 or more FPKM values were counted to be 25 genes in P1 and 31 genes in P7 (Fig 3B). About 50% CpBV genes among the highly expressed genes were common in parasitic stages and included E94K, CTL, CrV1, ELP, BEN, CST, and HP family members. However, some BEN, ELP, and HP genes were specific to early stage, while some PTP and SRP genes along with many HP genes were specifically highly expressed at late stage.

**Fig 4:**
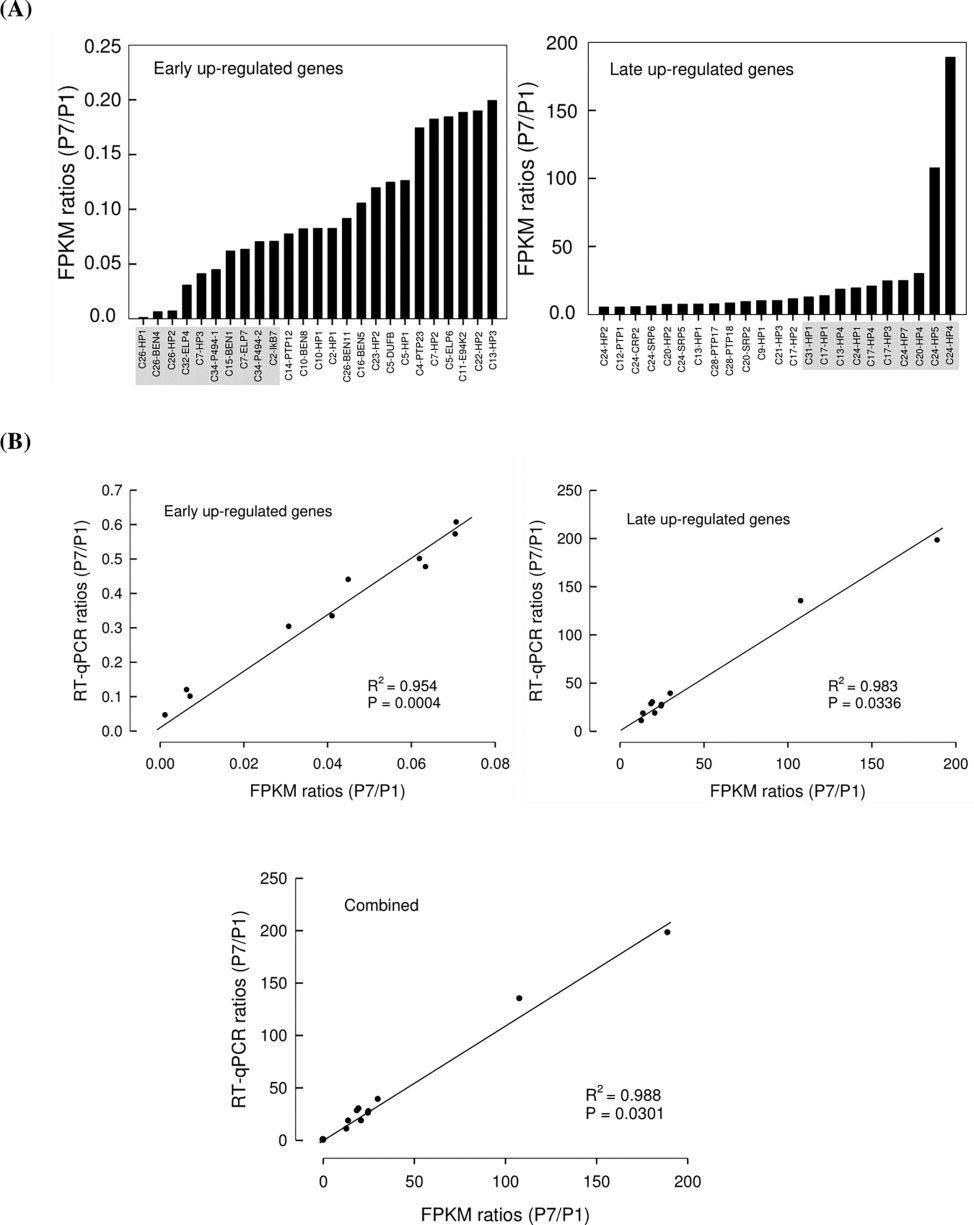
Regulation of CpBV gene expressions in *P*. *xylostella* during parasitism of *C*. *plutellae*. Regulation of gene expression was calculated by ratios of expression levels (FPKM values from Illumina HiSeq) at early (one days after parasitization: P1) and late (7 days after parasitization: P7) parasitic stages. (A) Early and late up-regulated genes. Shaded areas indicate top 10 up-regulated genes in each category. (B) Validation of expression levels obtained from Illumina HiSeq data (FPKM values) with relative transcripts levels obtained from RT-qPCR. Lines on spots represent linear regressions. ‘Combined’ indicate both early and late up-regulated genes.

To monitor expressional changes of CpBV ORFs during parasite development, FPKM ratios between P1 and P7 were assessed (Fig 4A). At early parasitic stage, BEN, ELP, P494, IkB, and PTP genes (shaded boxes in Fig 4A left panel) were highly induced by more than 15 folds than the late stage. Especially, *C26-HP1* was induced at early stage by 786 folds compared to late stage. In contrast, top 10 highly induced genes at late parasitic stage (shaded boxes in Fig 4A right panel) were all hypothetical genes, in which *C24-HP4* was induced by 189 folds compared to early stage. Top 10 highly expressed genes at early and late parasitic stages were validated by RT-qPCR (Fig 4B). This analysis indicated that there was more than 95% correlation between RNA-Seq and RT-qPCR.

**Fig 5:**
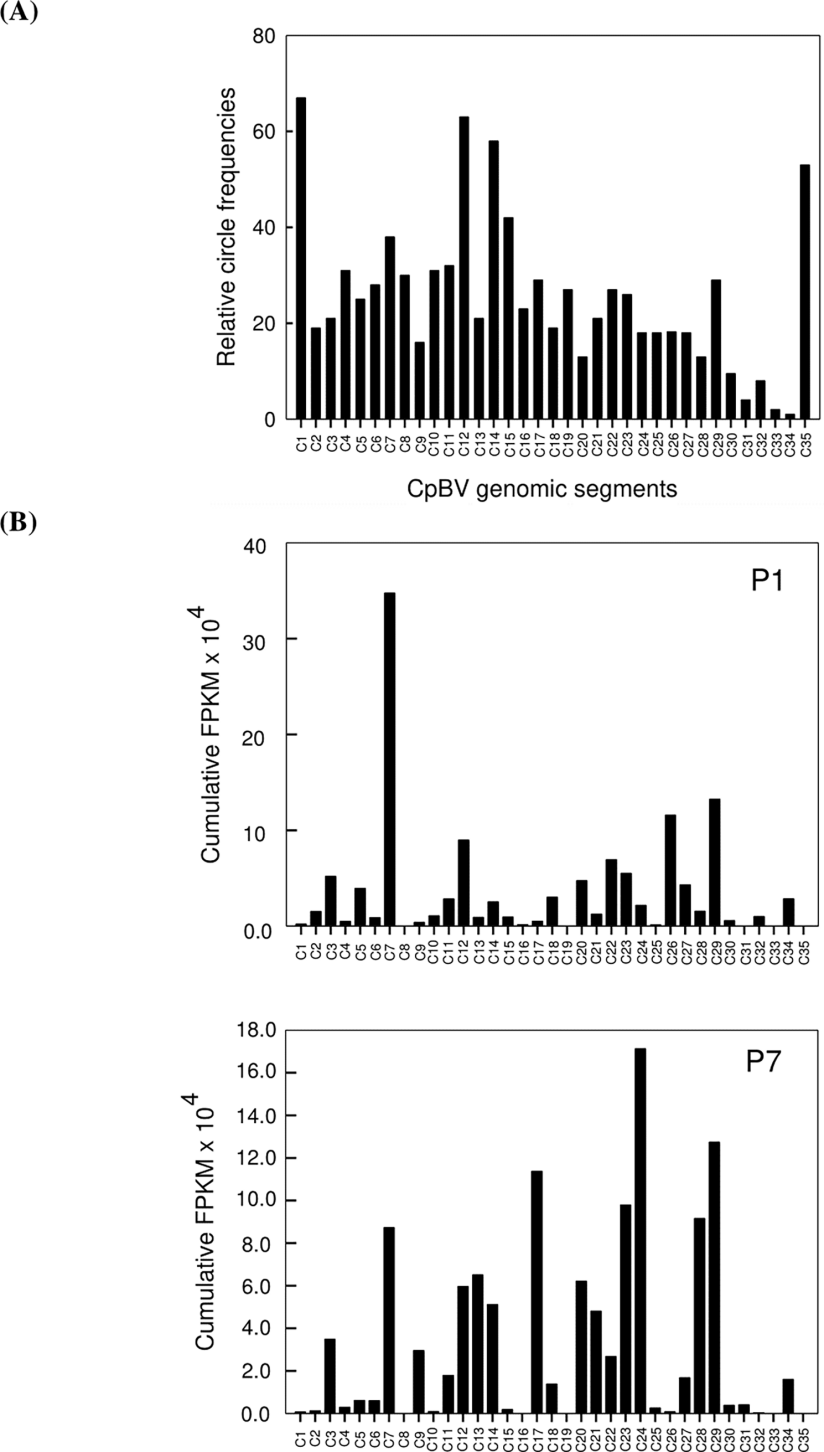
Relationship between frequencies of different CpBV genome circles in ovarian lumen and overall gene expression levels of the genome circles. (A) Relative frequencies of CpBV genome circles in ovarian lumen of *C*. *plutellae* [16] (B) Cumulative gene expression levels of different CpBV genome circles at early (one day after parasitization: P1) and late (7 days after parasitization: P7) parasitic stages. Cumulative gene expression levels were calculated by adding expression levels of all ORFs in individual CpBV genome circles.

To explain the differential expressions of CpBV genes according to relative frequencies of viral circles [16], FPKM values of different genes encoded in each viral circle were combined (Fig 5). There were significant differences between relative circle frequencies and gene expressions at P1 (*X*^*2*^ = 154,033; df = 34; *P* < 0.0001) or P7 (*X*^*2*^ = 156,963; df = 34; *P* < 0.0001) stages. In addition, gene expression levels of specific viral circles were also significantly different between P1 and P7 (*X*^*2*^ = 759,715; df = 31; *P* < 0.0001). These results indicated that there is little causal relationship between frequencies of CpBV genome circles and expression levels.

### 3.3. Change in gene expression levels of parasitized host

**Fig 6:**
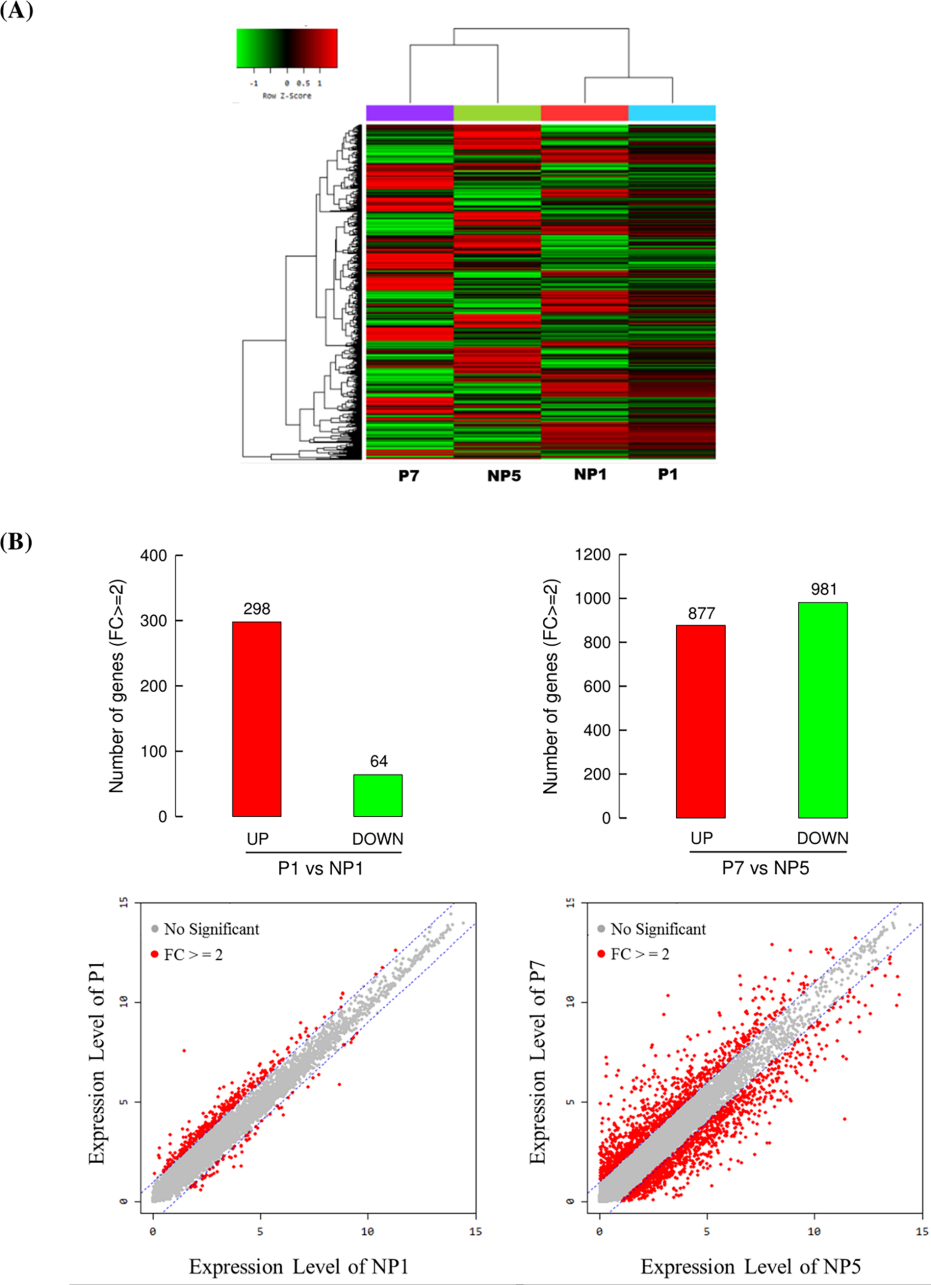
Change in host gene expression levels of *P*. *xylostella* parasitized (P) by *C*. *plutellae* at early (one day after parasitization: P1) and late (7 days after parasitization: P7) parasitic stages. In comparison, corresponding nonparasitized (NP) hosts were N1 stage for P1 and N5 stage for P7. (A) Clustering analysis of expression patterns. Heat-map analysis indicates two clusters of N1-P1 and N5-P7. Red color shows high expression levels while green color shows low expression levels. (B) Regulation of host gene expression by more than two fold change (FC) between P and NP. DEG analysis in lower panels indicates red spot genes that exhibit more than two fold change in gene expression levels between P1 and N1 (left panel) or P7 and N5 (right panel).

Most CpBV genes were expressed at early and late parasitic stages in parasitized host. This suggests that the parasitized host might undergo significant change in gene expression due to CpBV infection. To address this hypothesis, total transcriptomes at early and late parasitic stages were compared with those of nonparasitized hosts at the corresponding stages (Fig 6). A total of 18,073 transcripts were detected in both NP and P larvae by RNA-Seq analysis. For DEG analysis, 12,945 transcripts were used because 5,128 transcripts had 0 value in FPKM at least one sample among NP1, NP5, P1, and P7. Expression levels of these transcripts showed two distinct clusters of P7-NP5 and NP1-P1 (Fig 6A). At early parasitic stage, 362 (2.80%) transcripts exhibited more than two fold expression changes by parasitism (Fig 6B). At late parasitic stage, 1,858 (14.35%) transcripts exhibited more than two fold expression change.

These highly regulated genes were different between early and late parasitic stages (Table 2). At early (P1) stage, several enzymes such as esterase, lipase and trypsin were up-regulated along with unknown genes, while antimicrobial peptide and developmental genes (chitin-binding, JH-associated and zinc-finger proteins) were down-regulated in their expressions. At late stage, larval cuticle protein (LCP-30), some enzymes, and signal proteins were up-regulated, while pupal cuticle protein (LCP-17), storage protein, and other structural proteins were down-regulated.

**Table 2:** Top 10 highly regulated genes of *P*. *xylostella* parasitized by *C*. *plutellae* at early (P1) and late (P7) stages.

	Gene ID	Gene functions	Fold change
P1 vs N1 (up)	Px008247	Unknown function	68.6
	Px011756	Esterase FE4	5.9
	Px004988	Unknown function	5.8
	Px012011	Pancreatic triacylglycerol lipase	5.5
	Px016419	Unknown function	5.2
	Px017494	Unknown function	5.1
	Px005082	Unknown function	4.9
	Px000644	Pancreatic triacylglycerol lipase	4.8
	Px005241	Trypsin CFT-1	4.6
	Px007617	Trypsin CFT-1	4.3
P7 vs N5 (up)	Px005797	Insect intestinal mucin IIM14	144.6
	Px009655	Acyl-CoA Delta(11) desaturase	120.5
	Px002027	L-sorbose 1-dehydrogenase	96.1
	Px016045	Larval cuticle protein LCP-30	63.3
	Px001339	Larval cuticle protein LCP-30	63.3
	Px010162	Arp2	52.1
	Px003251	Putative cuticle protein	46.7
	Px004684	Tyrosine 3-monooxygenase	42.5
	Px012314	Death-associated small cytoplasmic leucine-rich protein	40.4
	Px010057	Urbain	29.6
P1 vs N1 (down)	Px013796	Hyphancin-3F	-6.9
	Px014502	Putative uncharacterized protein	-5.1
	Px009094	Zinc finger protein 445	-4.1
	Px008670	Unknown function	-3.3
	Px007023	Acidic juvenile hormone-suppressible protein 1	-3.1
	Px001431	Chitin binding PM protein	-3.1
	Px007182	Putative uncharacterized protein	-2.9
	Px011152	Unknown function	-2.9
	Px010298	Unknown function	-2.8
	Px012097	Unknown function	-2.7
P7 vs N5 (down)	Px011896	PAB-dependent poly(A)-specific ribonuclease subunit 2	-21.9
	Px014262	Putative cuticle protein	-25.3
	Px014406	Putative cuticle protein	-21.5
	Px014303	Vesicular glutamate transporter 1	-21.5
	Px009113	Glutathione S-transferase	-11.8
	Px017039	Putative acyl-CoA-binding protein	-11.8
	Px010466	Repetitive proline-rich cell wall protein 2	-16.7
	Px014247	Cuticular protein CPR77	-19.7
	Px001076	Fibrohexamerin	-10.2
	Px003253	Larval cuticle protein LCP-17	-10.8

## Discussion

CpBV genome is located on the host wasp chromosomes and has been considered to be composed of two parts [28]. Unencapsidated CpBV genome is likely originated from an ancestral nudivirus as demonstrated in other BV genomes [29] and predicted to play crucial roles in assisting viral replication and providing viral coat proteins. In contrast, encapsidated (ENC) CpBV genome encodes virulent factors to regulate physiological processes of parasitized host [30]. CpBV ENC genome was identified to encode 157 ORFs in 35 DNA circles (C1-C35) ranging from 2.6 to 39.2 kb [16]. CpBV ENC genome is similar to other PDV ENC genomes in high AT content, low coding gene density, and a large number of genes containing introns [16]. Half of ORFs were annotated into 13 eukaryotic conserved genes or gene families [16]. This study revised this annotation with updated gene database and recent functional studies related to BEN and E94K gene families [21,26]. Revised annotation of CpBV ENC genome comprises of viral/eukaryotic conserved families and hypothetical (HP) gene families. Compared to previous annotation [16], four families (P494, P325, E94K, SRP) are newly added. These four types of genes are known in other PDV genomes and named as specific gene families (see Fig 1). Especially, four E94K genes are homologous to *p94* gene of nucleopolyhedrosis virus and have been known in their physiological function to inhibit host immune and developmental processes [23]. However, RNaseT2 family in the previous study is now combined with the BEN family because several BEN family genes contain RNaseT2 domain [22]. From these revisions, the number of HP genes was reduced in CpBV ENC genome, in which 83 ORFs are now classified into 15 viral and eukaryotic conserved families, while the remaining 74 ORFs are in HP. In CcBV, HP genes are subdivided into different BV families depending on their sequence homology [7]. In CpBV, HP genes were not clearly separated in phylogenetic analysis to form distinct gene families. With additional functional study, HP genes of CpBV might be further subdivided.

Most of CpBV ENC genes appeared to be persistently expressed in parasitized host. Expression analysis was performed in parasitized host at two parasitic phases. Parasitized hosts at early (‘P1’) and late (‘P7’) parasitic phases expressed 145 and 143 CpBV ORFs out of 157 predicted ORFs. Surprisingly, 141 ORFs were expressed in both phases, indicating persistent expression nature of ENC CpBV genes. This CpBV expression pattern appears to be different with gene expression pattern of a highly similar ENC CcBV genome. ENC CcBV genome encodes 222 ORFs in 35 circles and expresses only 88 ORFs in parasitized host at early phase (24 after parasitization) [31]. These results indicate that 92.4% ORFs of CpBV were expressed in parasitized host, while 39.6% ORFs of CcBV were expressed in similar parasitic stage. This difference in the percentage of genes expressed in parasitized host between the two congener PDVs may be explained by different hosts: *P*. *xylostella* and *M*. *sexta*. Also, the analyzed tissues were different between two assessments, in which CcBV transcripts were assessed only from hemocytes and fat body, but CpBV transcripts were taken from whole body. In addition, sequencing depth of transcripts may be another factor. Expression of CcBV genes in parasitized host was analyzed by 454 pyrosequencing and gave 111,959 reads in both fat body and hemocyte tissues of parasitized host, while CpBV transcripts were assessed by Illumina HiSeq and resulted in 520,544,636 reads. Thus, genes expressed at relatively low levels can be identified in the current CpBV expression analysis.

There was a high variation in expression levels among different ENC CpBV genes. Among highly expressed genes (FPKM > 10,000), almost 50% genes kept their high expression levels at both P1 and P7. Chen et al. [16] showed unequal numbers of CpBV viral circles in the ovarian lumen, in which CpBV replicated in the ovarian calyx cells is accumulated. Thus, high number of replicated DNA circles may result in high expression levels in their encoding genes. However, though high frequencies of C1, C12, C14 and C35 CpBV DNAs were detected in ovarian calyx lumen, their encoding genes did not show high expression levels at both P1 and P7. Lack of relationship between relative frequencies of CpBV DNA circles and expression levels of their encoding genes was supported by statistical analyses of their independency. On the other hand, expression of each CpBV gene appeared to be regulated by interaction with host physiological status. Comparison in expression levels of CpBV genes between early (P1) and late (P7) stages showed that *C26-HP1* decreased about 786 folds in late stage, while *C24-HP4* increased about 189 folds in late stage. Thus, CpBV gene expression appeared to be dependent on host developmental status. Interestingly, CpBV genes classified into eukaryotic conserved gene families such as BEN, ELP, P494, IkB, and PTP were highly induced at early parasitic phase. In contrast, HP genes were highly induced in their expression at late stage. Some CpBV genes classified into BEN, ELP, IkB and PTP families have been known to inhibit host immune responses [21,28,32,33]. Thus, at early parasitic stage, immunosuppressive genes appear to be induced in their expression. The physiological functions of HP genes at late stage are not known. However, they might be associated with host regulation by inducing developmental retardation because *P*. *xylostella* larvae parasitized by *C*. *plutellae* extend their last instar by about 2 days at 25°C [15] and CpBV-infected larvae exhibit such extension of larval period [28]. In CcBV, gene expression analysis in parasitized host suggested that several factors, such as presence of signal peptides in encoded proteins, diversification of promoter regions, and gene position on the proviral genome influence on regulation of the viral gene expression [31]. The last factor is related with frequency of replicated viral circles because viral gene position on the proviral genome appears to determine copy number of replicated circles of CcBV [7]. As discussed earlier, CpBV circle frequency in the ovarian lumen is not likely to be associated with variation of the viral gene expression levels. Alternatively, individual viral gene characters, such as their promoters or secretory nature of the viral proteins [31] are likely to play crucial roles in determining the viral gene expression levels. Indeed, highly expressed CpBV genes at P1 included secretory proteins such as ELP, CrV1, CST, and CTL. Furthermore, mRNA stability raised by Beck et al. [34] may be another factor to determine transcript levels of the viral genes.

Host gene expression was highly influenced by parasitism of *C*. *plutellae* along with CpBV gene expression. In addition, the regulation of host gene expression was varied in different parasitic stages. At early stage (P1), 362 host genes exhibited more than two fold changes in expression levels compared to nonparasitized host at equivalent age. However, at late stage (P7), 1,858 host genes exhibited more than two fold changes. Based on the most highly changed 10 genes, host at early parasitic stage up-regulated expression of esterase, lipase, and trypsin genes, but down-regulated hyphancin, zinc-finger protein, JH-associated protein, and chitin-binding protein. Hyphancin is an antifungal peptide isolated from *Hyphantria cunea* [35]. In a related study, *M*. *sexta* parasitized by *C*. *congregata* did not show much suppression in gene expressions in antimicrobial peptides, though there were significant decreases in gene expressions related with cellular immunity [36]. These suggest that parasitism stimulates genes associated with host digestion, but inhibits immune and developmental genes. At late stage, gene expression of larval cuticle protein (LCP-30) [37] was up-regulated, but those of pupal cuticle protein (LCP-17) [38] and storage protein were down-regulated. Thus, developmental genes may be regulated by parasitism of *C*. *plutellae*. In this study, it is difficult to make any causative link between CpBV and host target genes. However, our data support the host regulation in different parasitic stages by differential expression of CpBV genes, in which several viral genes known to suppress host immunity were highly up-regulated in expression at P1. In contrast, highly up-regulated genes at P7 were hypothetical in function. Thus the differential regulation of host cuticle protein genes suggests that the HP genes may have a function in regulating host development.

In summary, most CpBV genes in viral particles are expressed in parasitized host. However, their expression levels vary with different parasitic stages. Our data on alterations of host gene expression in parasitized larvae suggest that the differential expressions of CpBV genes contribute to regulate host physiological processes.

## Supporting information

S1 Fig Phylogenetic analysis of protein tyrosine phosphatase (PTP) genes in parasitized *P*. *xylostella*. Amino acid sequences of these genes were retrieved from GenBank with accession numbers listed in S3 Table. Amino acid sequences were aligned with MEGA6 (Tamura et al., 2011). Bootstrap values on branches were obtained with 500 repetitions. The black box indicates the conserved regions among the genes. Red symbol indicates PTP catalytically active site.
(DOC)

S2 Fig Phylogenetic analysis of BANP, E5R and NAC1 (BEN) genes in parasitized *P*. *xylostella*. Amino acid sequences of these genes were retrieved from GenBank with accession numbers listed in S3 Table. Amino acid sequences were aligned with MEGA6 (Tamura et al., 2011). Bootstrap values on branches were obtained with 500 repetitions. The black box indicates the conserved regions among the genes.
(DOC)

S3 Fig Phylogenetic analysis of Inhibitor kB-like (IkB) genes in parasitized *P*. *xylostella*. Amino acid sequences of these genes were retrieved from GenBank with accession numbers listed in S3 Table. Amino acid sequences were aligned with MEGA6 (Tamura et al., 2011). Bootstrap values on branches were obtained with 500 repetitions. The black box indicates the conserved regions among the genes.
(DOC)

S4 Fig Phylogenetic analysis of EP1-like (ELP) genes in parasitized *P*. *xylostella*. Amino acid sequences of these genes were retrieved from GenBank with accession numbers listed in S3 Table. Amino acid sequences were aligned with MEGA6 (Tamura et al., 2011). Bootstrap values on branches were obtained with 500 repetitions. The black box indicates the conserved regions among the genes.
(DOC)

S5 Fig Phylogenetic analysis of serine-rich protein (SRP) genes in parasitized *P*. *xylostella*. Amino acid sequences of these genes were retrieved from GenBank with accession numbers listed in S3 Table. Amino acid sequences were aligned with MEGA6 (Tamura et al., 2011). Bootstrap values on branches were obtained with 500 repetitions. The black box indicates the conserved regions among the genes.
(DOC)

S6 Fig Phylogenetic analysis of E94K genes in parasitized *P*. *xylostella*. Amino acid sequences of these genes were retrieved from GenBank with accession numbers listed in S3 Table. Amino acid sequences were aligned with MEGA6 (Tamura et al., 2011). Bootstrap values on branches were obtained with 500 repetitions. The black box indicates the conserved regions among the genes.
(DOC)

S7 Fig Phylogenetic analysis of cysteine-rich proteins (CRP) genes in parasitized *P*. *xylostella*. Amino acid sequences of these genes were retrieved from GenBank with accession numbers listed in S3 Table. Amino acid sequences were aligned with MEGA6 (Tamura et al., 2011). Bootstrap values on branches were obtained with 500 repetitions. The black box indicates the conserved regions among the genes.
(DOC)

S8 Fig Phylogenetic analysis of C-type lectins (CTL) genes in parasitized *P*. *xylostella*. Amino acid sequences of these genes were retrieved from GenBank with accession numbers listed in S3 Table. Amino acid sequences were aligned with MEGA6 (Tamura et al., 2011). Bootstrap values on branches were obtained with 500 repetitions. The black box indicates the conserved regions among the genes.
(DOC)

S9 Fig Phylogenetic analysis of P494 genes in parasitized *P*. *xylostella*. Amino acid sequences of these genes were retrieved from GenBank with accession numbers listed in S3 Table. Amino acid sequences were aligned with MEGA6 (Tamura et al., 2011). Bootstrap values on branches were obtained with 500 repetitions. The black box indicates the conserved regions among the genes.
(DOC)

S10 Fig Phylogenetic analysis of *Cotesia rubecula* bracovirus (CrV1) genes in parasitized *P*. *xylostella*. Amino acid sequences of these genes were retrieved from GenBank with accession numbers listed in S3 Table. Amino acid sequences were aligned with MEGA6 (Tamura et al., 2011). Bootstrap values on branches were obtained with 500 repetitions. The black box indicates the conserved regions among the genes.
(DOC)

S11 Fig Phylogenetic analysis of cystatin (CST) genes in parasitized *P*. *xylostella*. Amino acid sequences of these genes were retrieved from GenBank with accession numbers listed in S3 Table. Amino acid sequences were aligned with MEGA6 (Tamura et al., 2011). Bootstrap values on branches were obtained with 500 repetitions. The black box indicates the conserved regions among the genes.
(DOC)

S12 Fig Phylogenetic analysis of viral histone H4 (vH4) genes in parasitized *P*. *xylostella*. Amino acid sequences of these genes were retrieved from GenBank with accession numbers listed in S3 Table. Amino acid sequences were aligned with MEGA6 (Tamura et al., 2011). Bootstrap values on branches were obtained with 500 repetitions. The black box indicates the conserved regions among the genes.
(DOC)

S13 Fig Phylogenetic analysis of duffy binding-like domain (DUFB) genes in parasitized *P*. *xylostella*. Amino acid sequences of these genes were retrieved from GenBank with accession numbers listed in S3 Table. Amino acid sequences were aligned with MEGA6 (Tamura et al., 2011). Bootstrap values on branches were obtained with 500 repetitions. The black box indicates the conserved regions among the genes.
(DOC)

S14 Fig Phylogenetic analysis of DNA helicase (dHEL) genes in parasitized *P*. *xylostella*. Amino acid sequences of these genes were retrieved from GenBank with accession numbers listed in S3 Table. Amino acid sequences were aligned with MEGA6 (Tamura et al., 2011). Bootstrap values on branches were obtained with 500 repetitions. The black box indicates the conserved regions among the genes.
(DOC)

S15 Fig Phylogenetic analysis of P325 genes in parasitized *P*. *xylostella*. Amino acid sequences of these genes were retrieved from GenBank with accession numbers listed in S3 Table. Amino acid sequences were aligned with MEGA6 (Tamura et al., 2011). Bootstrap values on branches were obtained with 500 repetitions. The black box indicates the conserved regions among the genes.
(DOC)

S16 Fig Phylogenetic analyses of all 74 hypothetical (HP) ORFs. Amino acid sequences of these genes were retrieved from GenBank with accession numbers listed in S3 Table. Amino acid sequences were aligned with MEGA6 (Tamura et al., 2011). Bootstrap values on branches were obtained with 500 repetitions.
(DOC)

S1 Table RNA-Seq summary of *P*. *xylostella* gene expression in nonparasitized (NP) and parasitized (P). (DOC)

S2 Table List of primers used in RT-PCR and RT-qPCR. (DOC)

S3 Table List of NCBI-GenBank accession numbers used in phylogenetic analyses. (DOC)
