# Supplementary figures and images for: Persistent expression of Cotesia plutellae bracovirus genes in parasitized host, Plutella xylostella
Source: PLoS One. 2018 Jul 16;13(7):e0200663. doi: 10.1371/journal.pone.0200663 (PMC6047808; doi:10.1371/journal.pone.0200663)

**
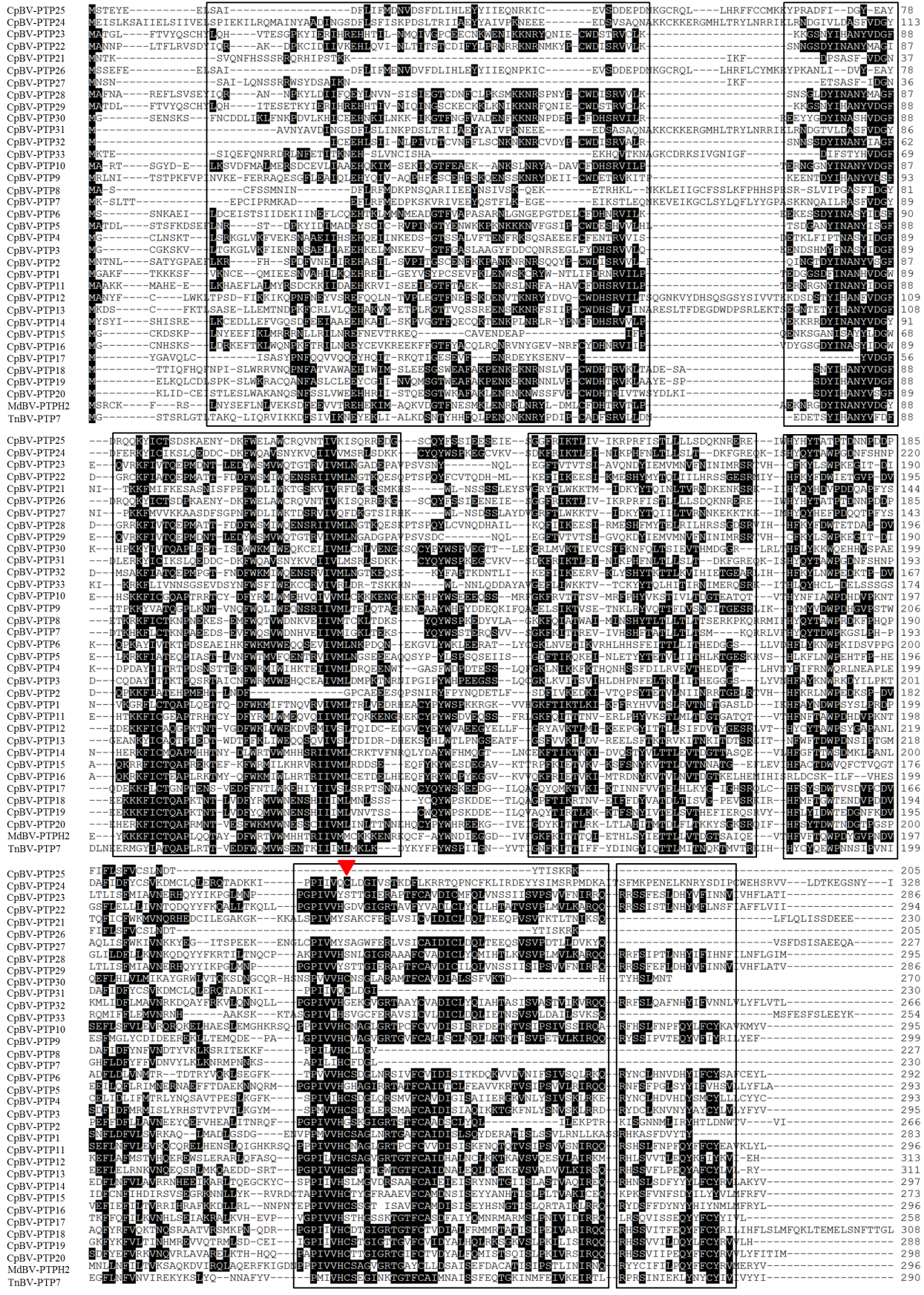
**

Supplement: S1 Fig — Amino acid sequences of these genes were retrieved from GenBank with accession numbers listed in S3 Table. Amino acid sequences were aligned with MEGA6 (Tamura et al., 2011). Bootstrap values on branches were obtained with 500 repetitions. The black box indicates the conserved regions among the genes. Red symbol indicates PTP catalytically active site. (DOC) [file pone.0200663.s001.doc]

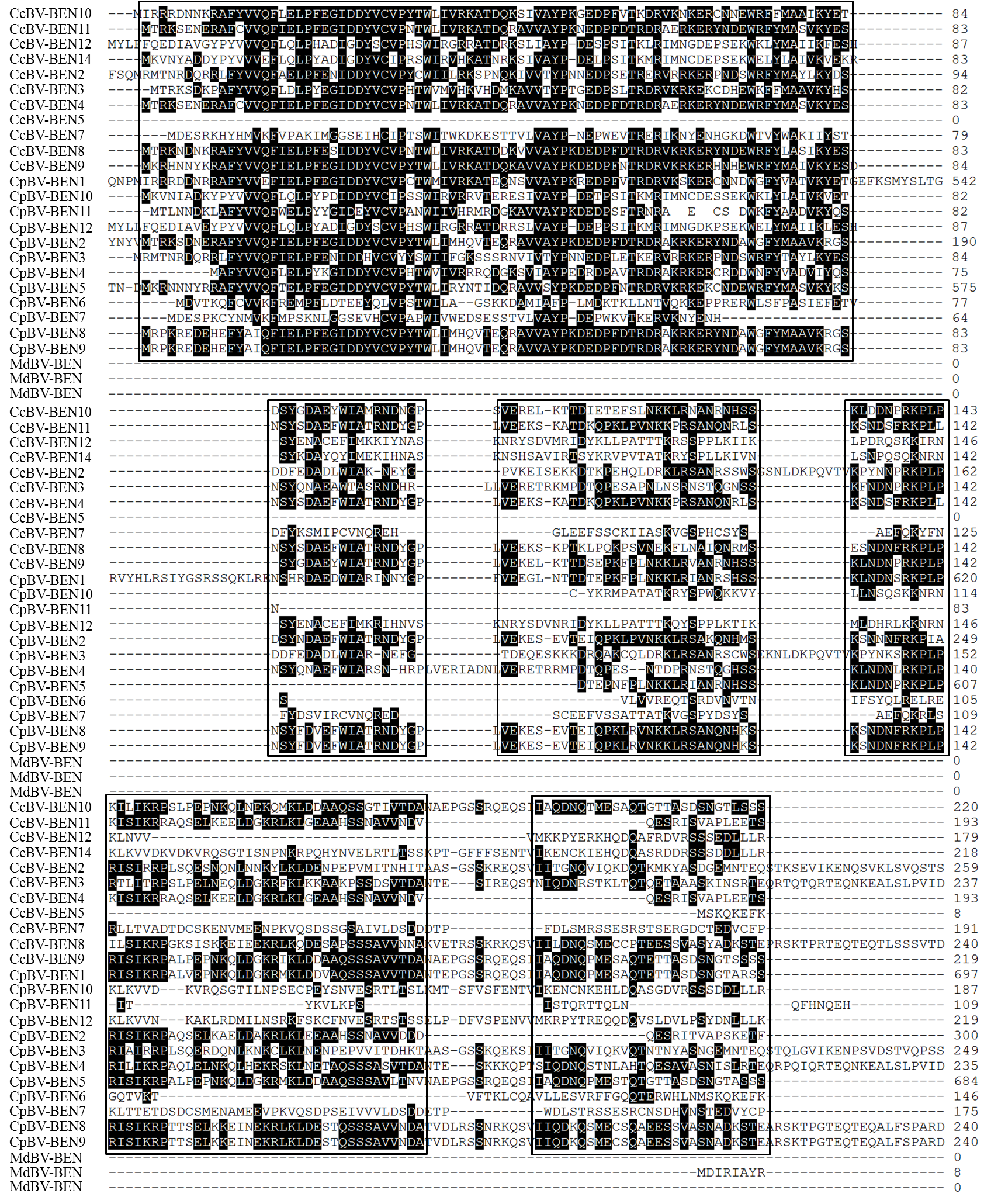


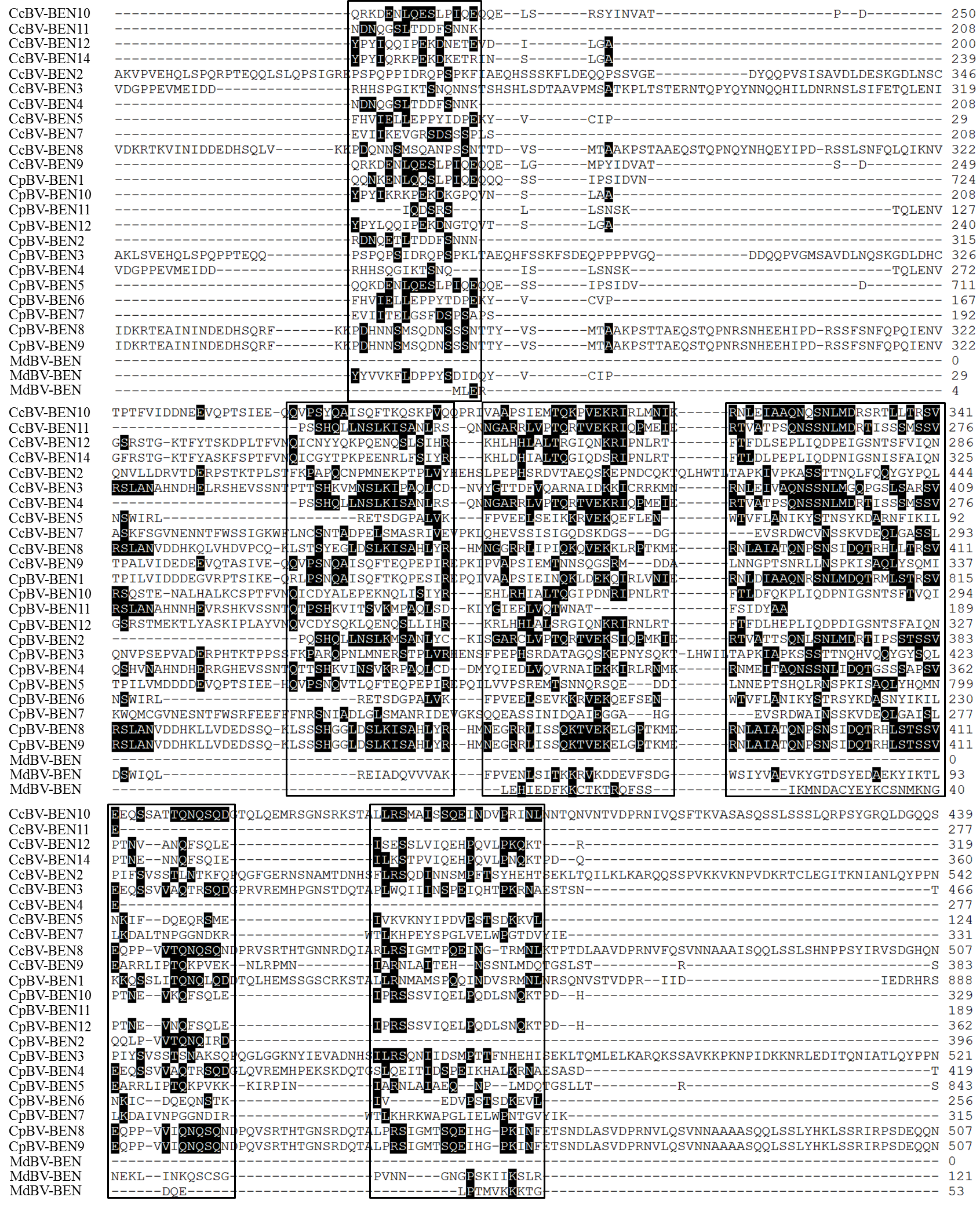


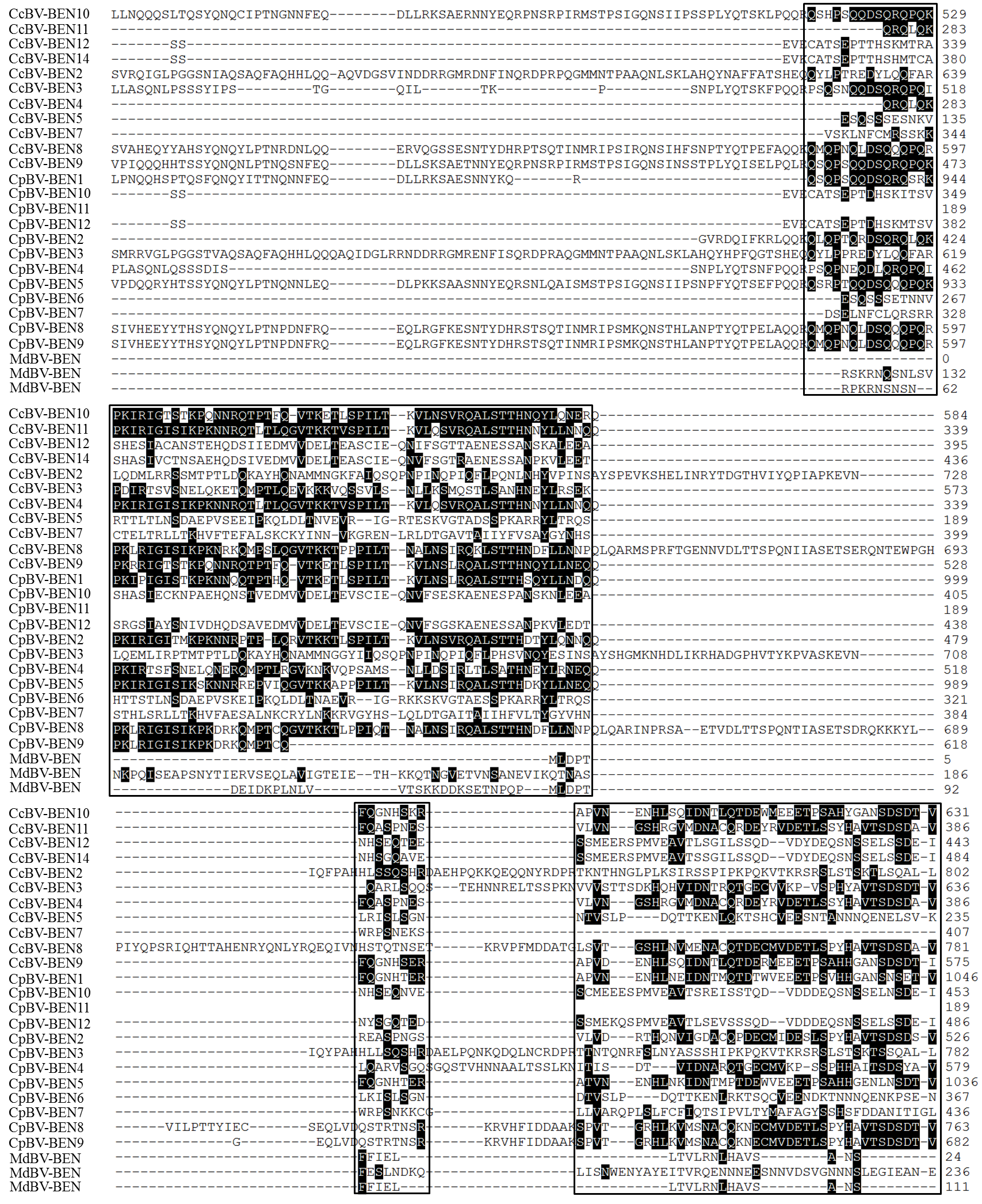


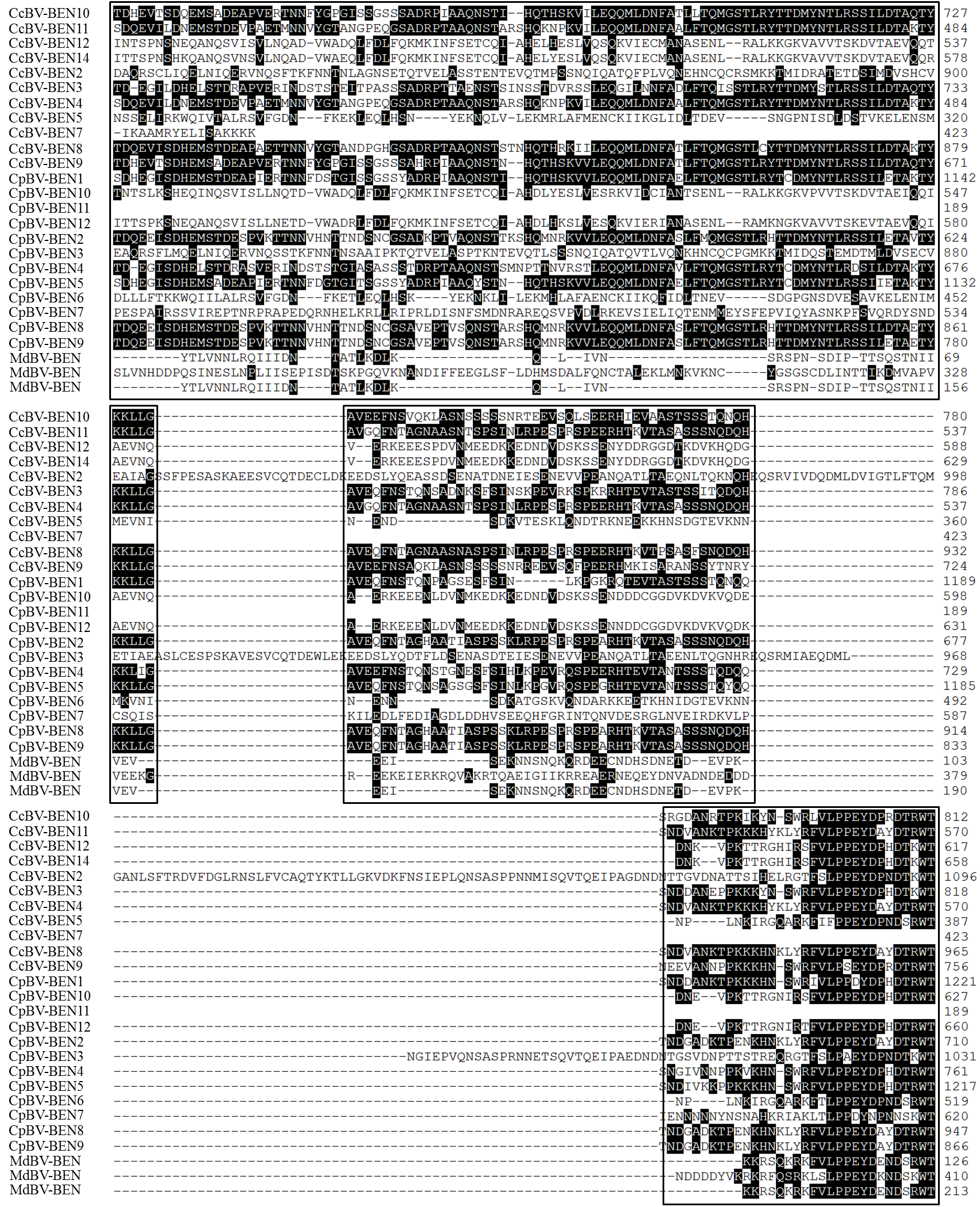


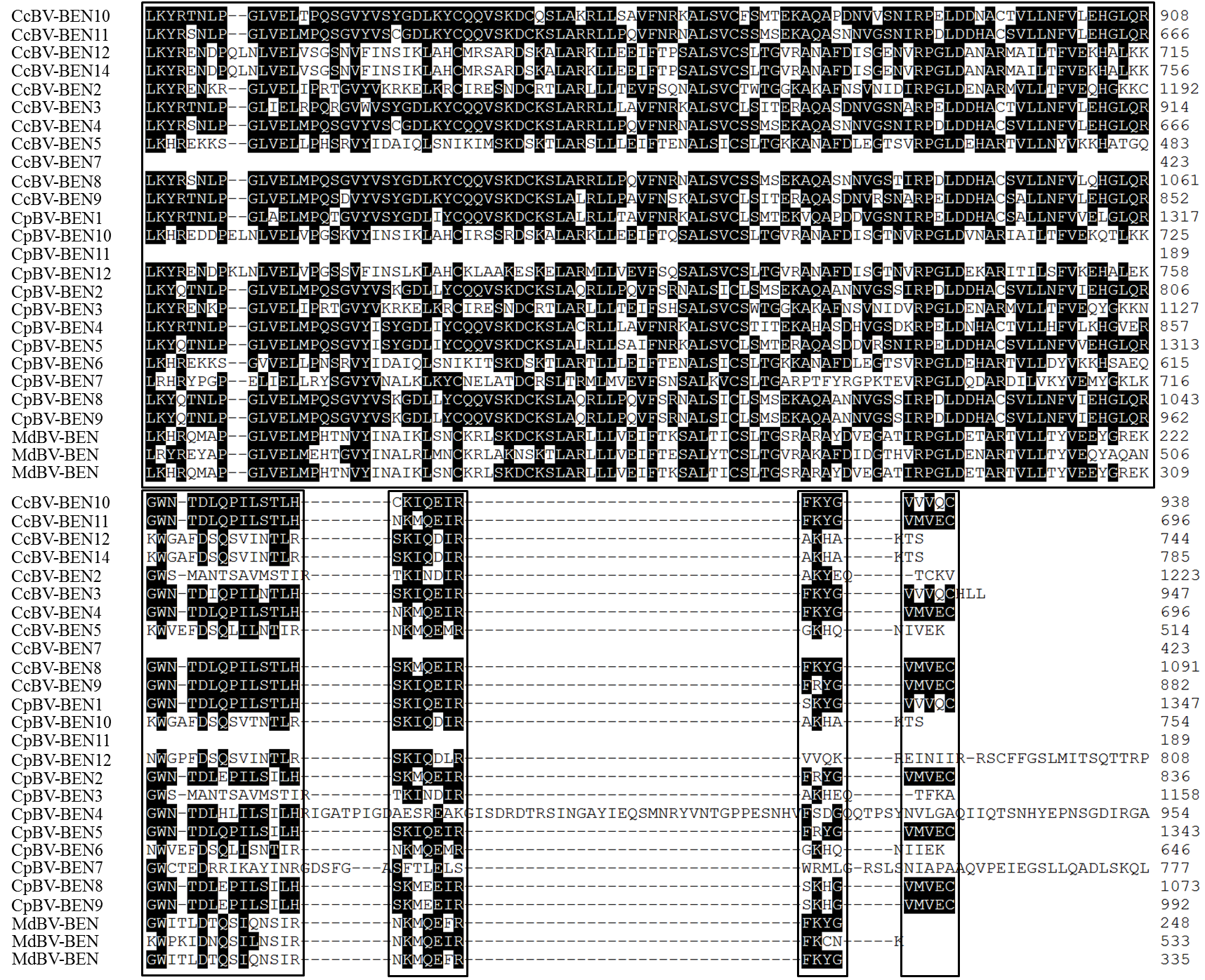


**S2 Fig**

Supplement: S2 Fig — Amino acid sequences of these genes were retrieved from GenBank with accession numbers listed in S3 Table. Amino acid sequences were aligned with MEGA6 (Tamura et al., 2011). Bootstrap values on branches were obtained with 500 repetitions. The black box indicates the conserved regions among the genes. (DOC) [file pone.0200663.s002.doc]

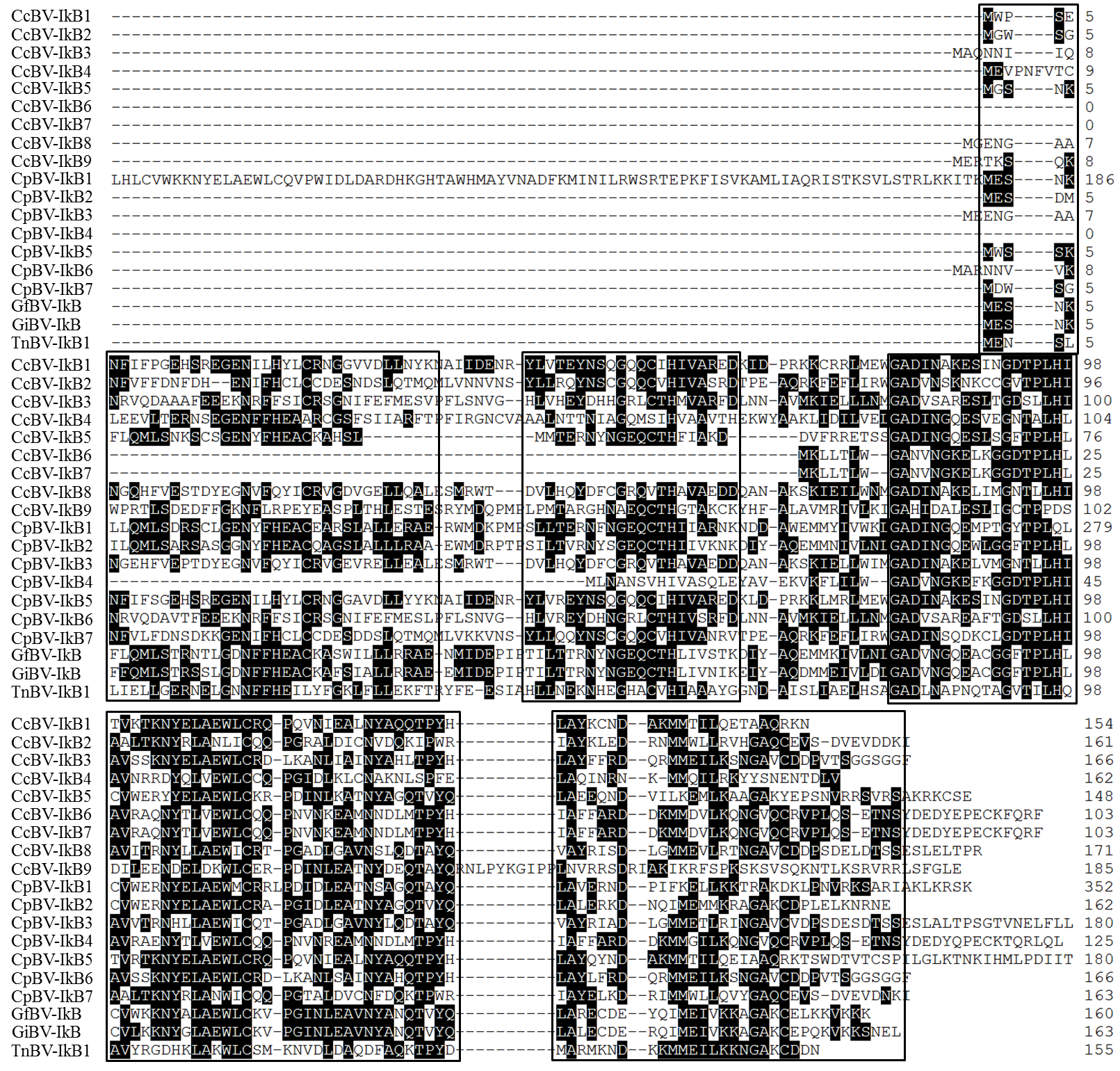

Supplement: S3 Fig — Amino acid sequences of these genes were retrieved from GenBank with accession numbers listed in S3 Table. Amino acid sequences were aligned with MEGA6 (Tamura et al., 2011). Bootstrap values on branches were obtained with 500 repetitions. The black box indicates the conserved regions among the genes. (DOC) [file pone.0200663.s003.doc]

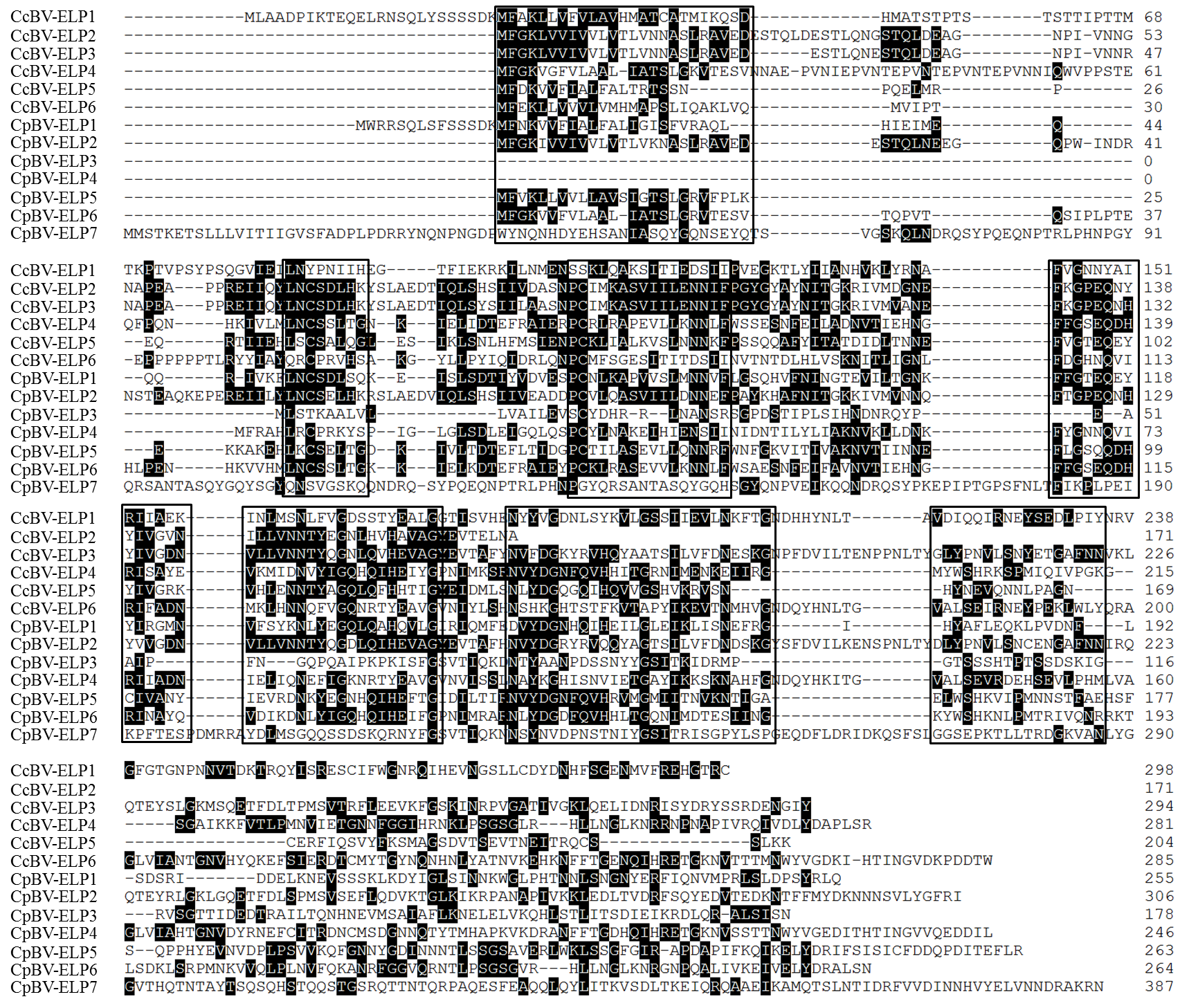

Supplement: S4 Fig — Amino acid sequences of these genes were retrieved from GenBank with accession numbers listed in S3 Table. Amino acid sequences were aligned with MEGA6 (Tamura et al., 2011). Bootstrap values on branches were obtained with 500 repetitions. The black box indicates the conserved regions among the genes. (DOC) [file pone.0200663.s004.doc]

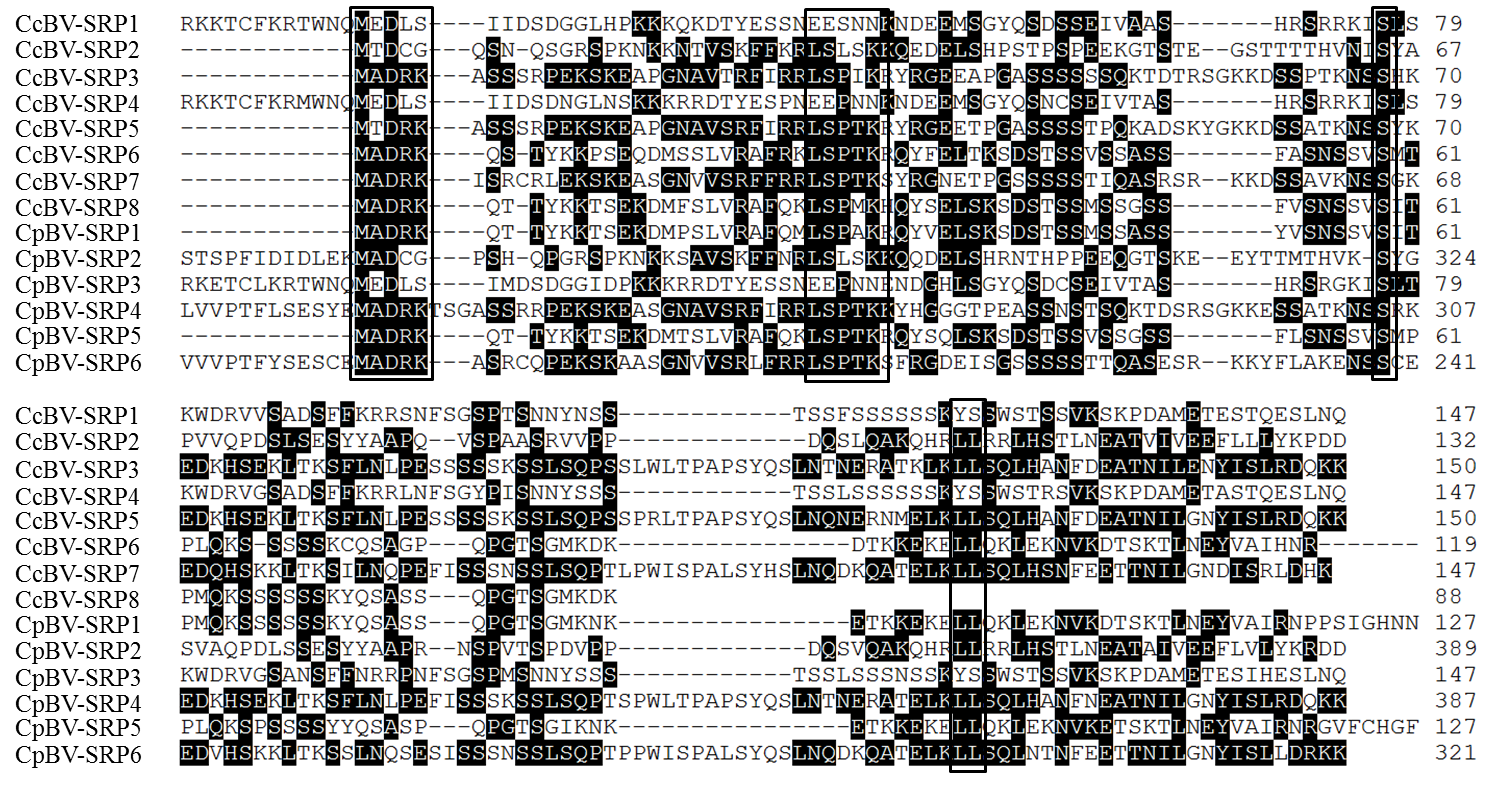

Supplement: S5 Fig — Amino acid sequences of these genes were retrieved from GenBank with accession numbers listed in S3 Table. Amino acid sequences were aligned with MEGA6 (Tamura et al., 2011). Bootstrap values on branches were obtained with 500 repetitions. The black box indicates the conserved regions among the genes. (DOC) [file pone.0200663.s005.doc]

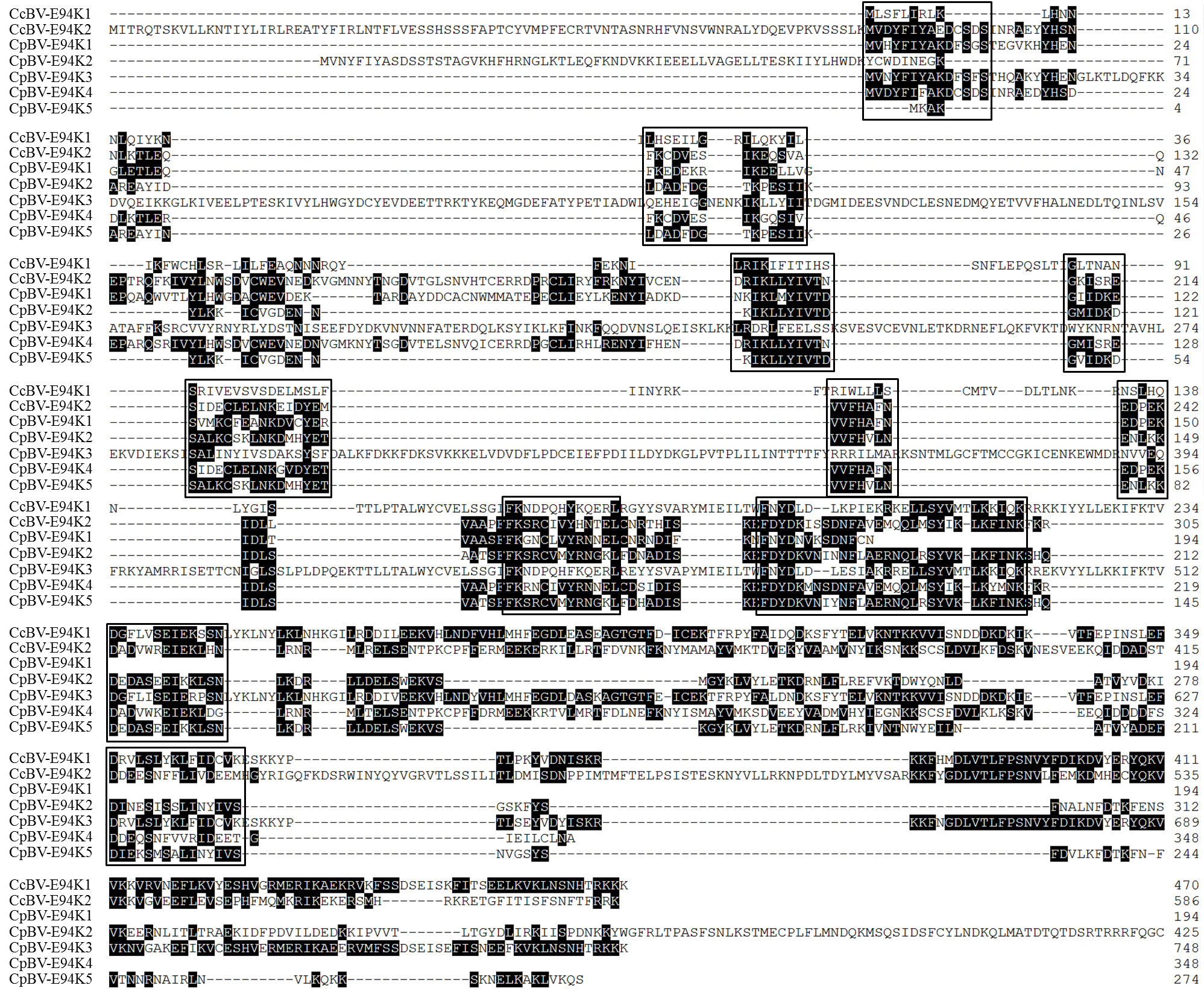

Supplement: S6 Fig — Amino acid sequences of these genes were retrieved from GenBank with accession numbers listed in S3 Table. Amino acid sequences were aligned with MEGA6 (Tamura et al., 2011). Bootstrap values on branches were obtained with 500 repetitions. The black box indicates the conserved regions among the genes. (DOC) [file pone.0200663.s006.doc]

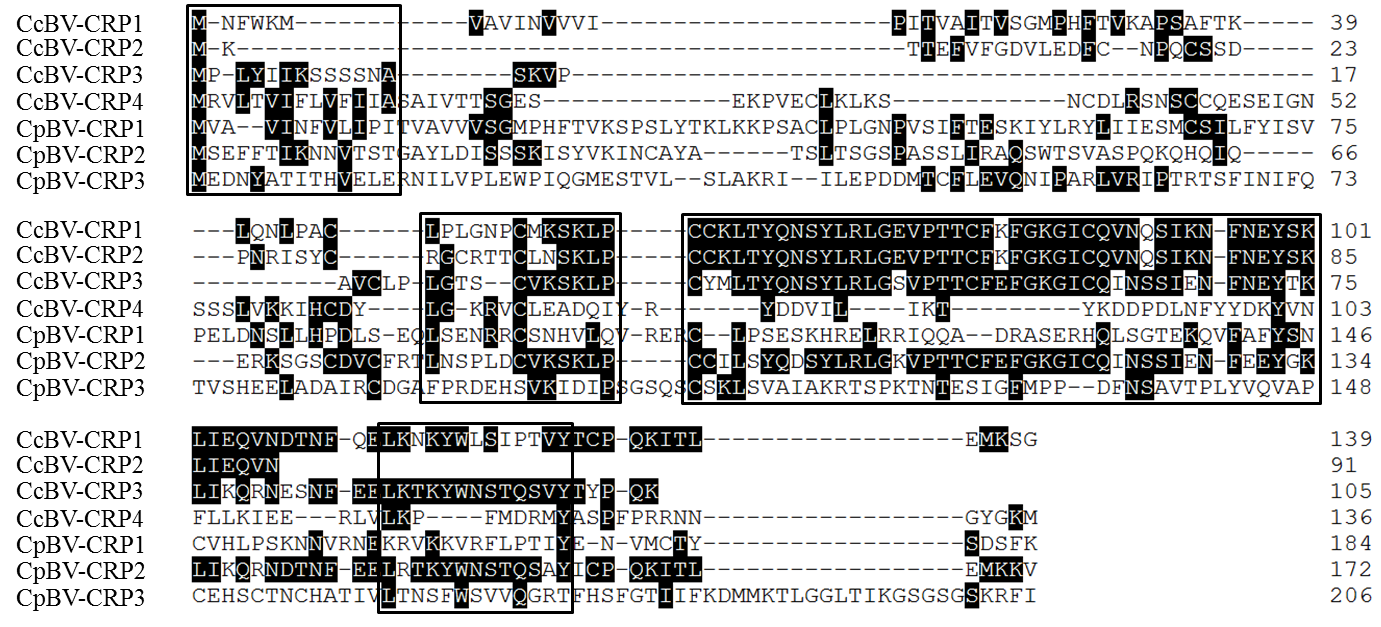

Supplement: S7 Fig — Amino acid sequences of these genes were retrieved from GenBank with accession numbers listed in S3 Table. Amino acid sequences were aligned with MEGA6 (Tamura et al., 2011). Bootstrap values on branches were obtained with 500 repetitions. The black box indicates the conserved regions among the genes. (DOC) [file pone.0200663.s007.doc]

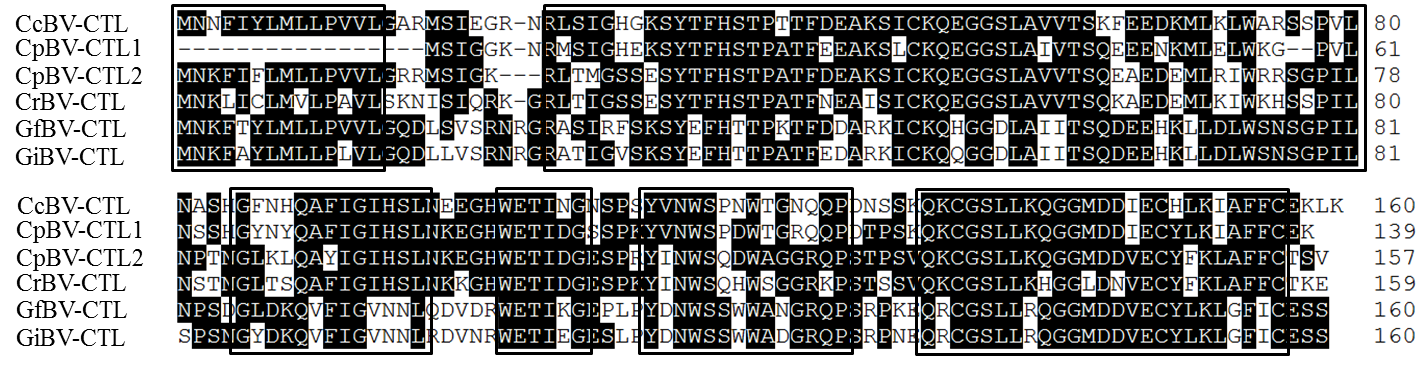

Supplement: S8 Fig — Amino acid sequences of these genes were retrieved from GenBank with accession numbers listed in S3 Table. Amino acid sequences were aligned with MEGA6 (Tamura et al., 2011). Bootstrap values on branches were obtained with 500 repetitions. The black box indicates the conserved regions among the genes. (DOC) [file pone.0200663.s008.doc]

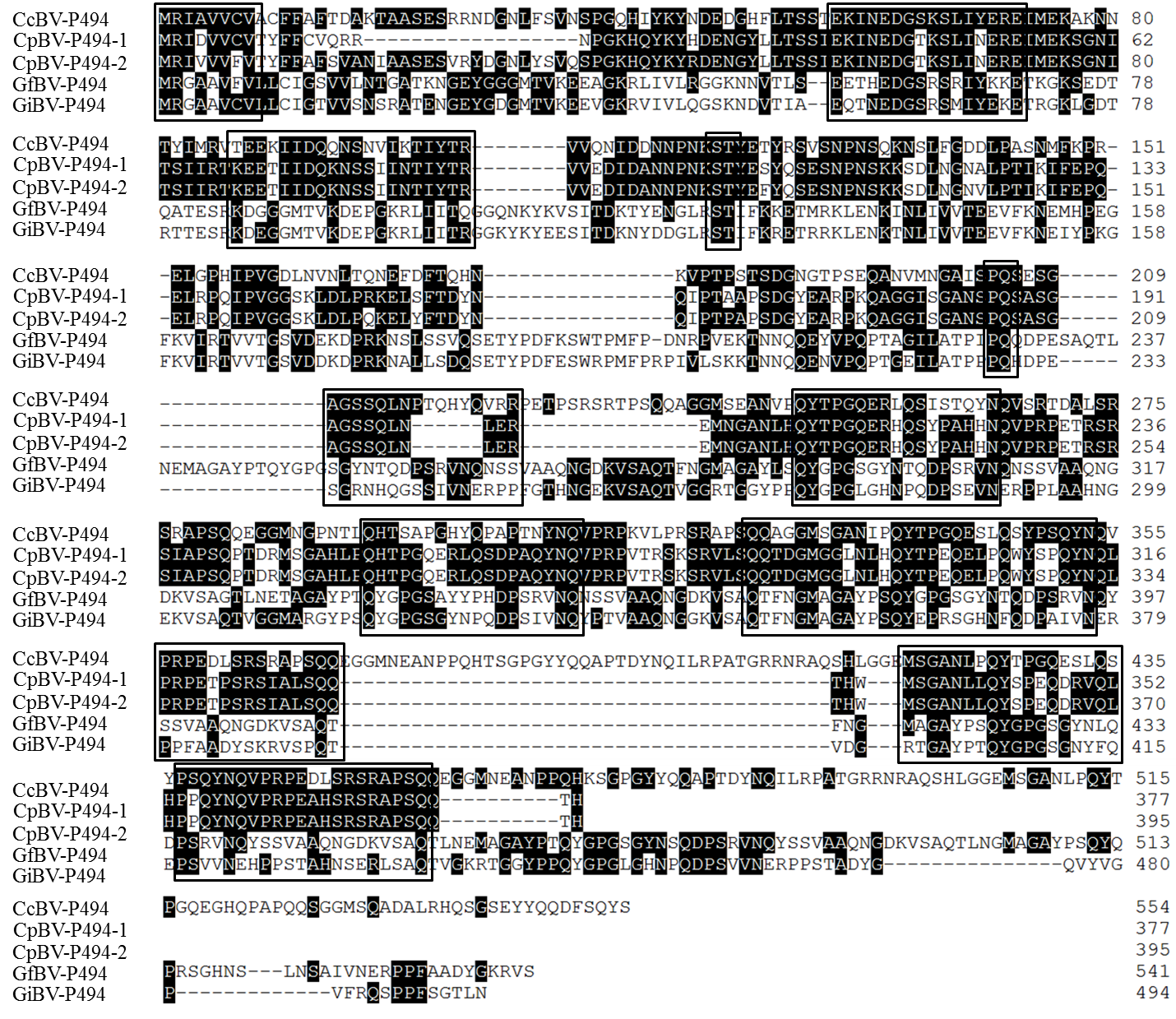

Supplement: S9 Fig — Amino acid sequences of these genes were retrieved from GenBank with accession numbers listed in S3 Table. Amino acid sequences were aligned with MEGA6 (Tamura et al., 2011). Bootstrap values on branches were obtained with 500 repetitions. The black box indicates the conserved regions among the genes. (DOC) [file pone.0200663.s009.doc]

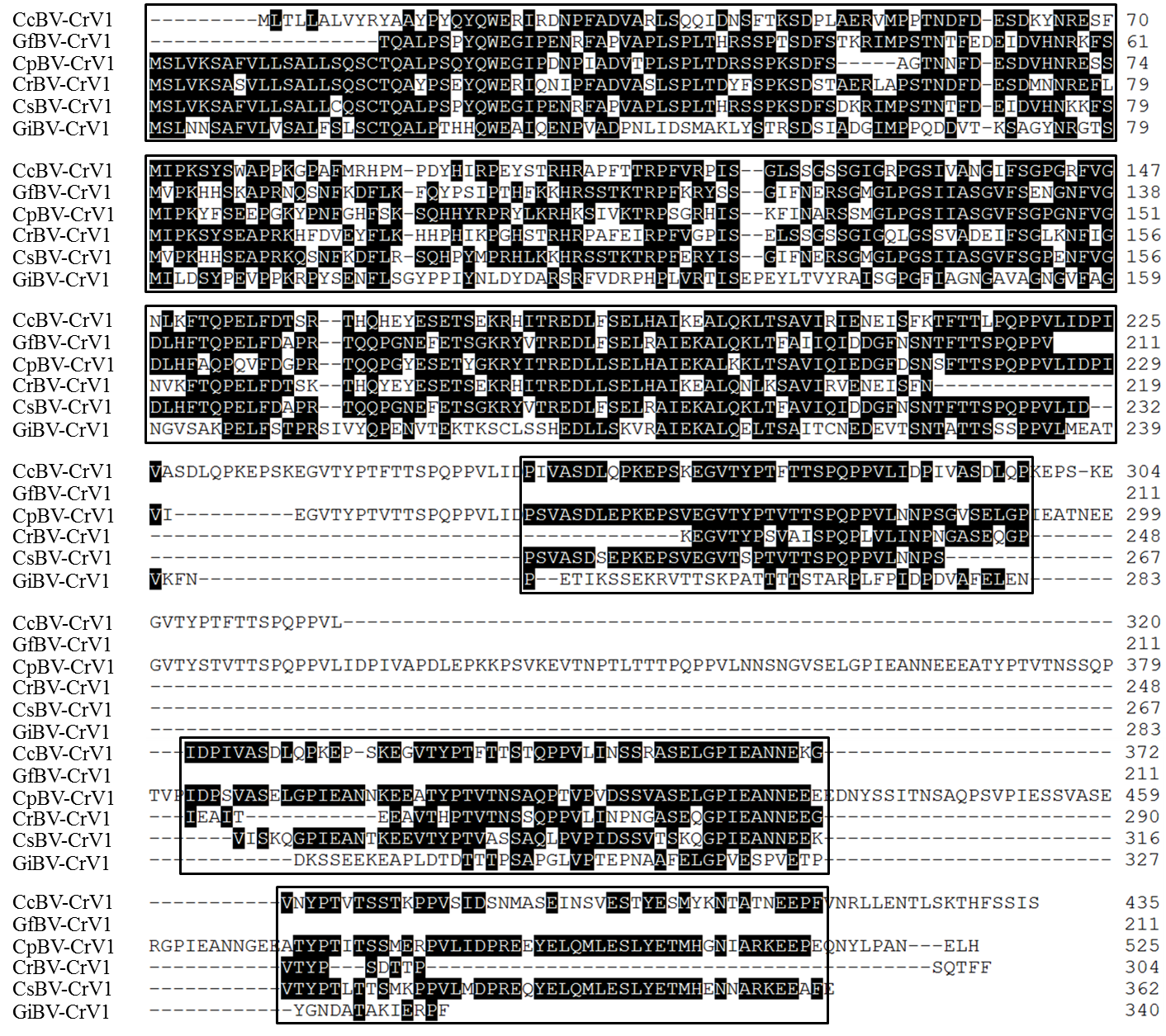

Supplement: S10 Fig — Amino acid sequences of these genes were retrieved from GenBank with accession numbers listed in S3 Table. Amino acid sequences were aligned with MEGA6 (Tamura et al., 2011). Bootstrap values on branches were obtained with 500 repetitions. The black box indicates the conserved regions among the genes. (DOC) [file pone.0200663.s010.doc]

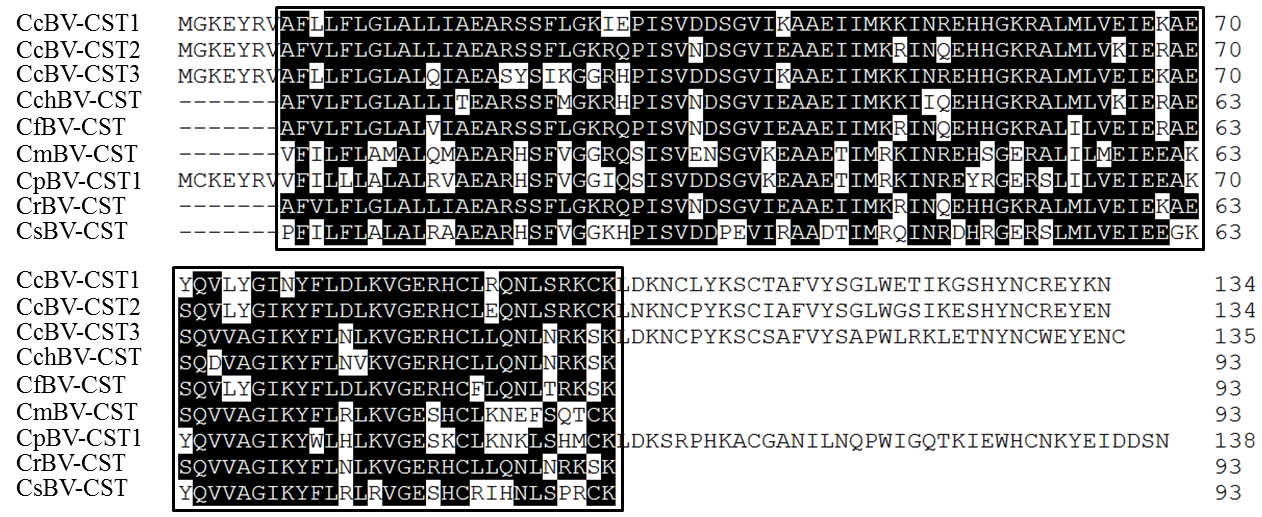

Supplement: S11 Fig — Amino acid sequences of these genes were retrieved from GenBank with accession numbers listed in S3 Table. Amino acid sequences were aligned with MEGA6 (Tamura et al., 2011). Bootstrap values on branches were obtained with 500 repetitions. The black box indicates the conserved regions among the genes. (DOC) [file pone.0200663.s011.doc]

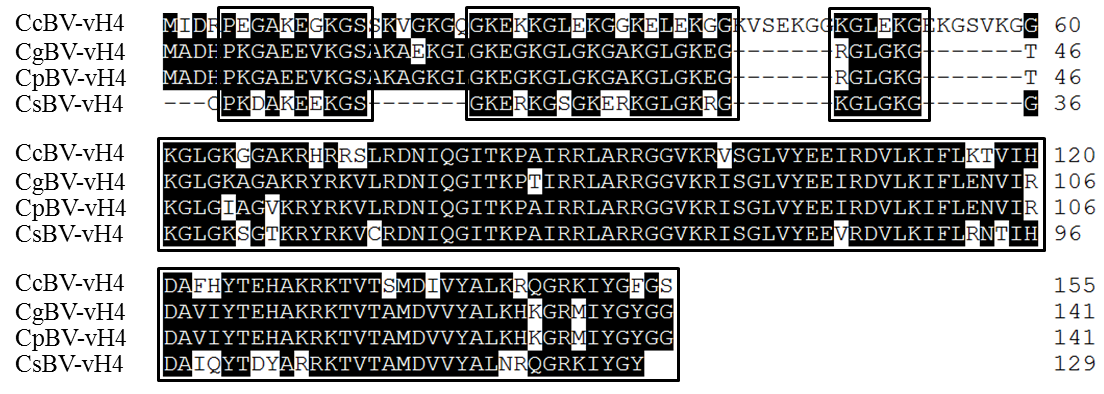

Supplement: S12 Fig — Amino acid sequences of these genes were retrieved from GenBank with accession numbers listed in S3 Table. Amino acid sequences were aligned with MEGA6 (Tamura et al., 2011). Bootstrap values on branches were obtained with 500 repetitions. The black box indicates the conserved regions among the genes. (DOC) [file pone.0200663.s012.doc]

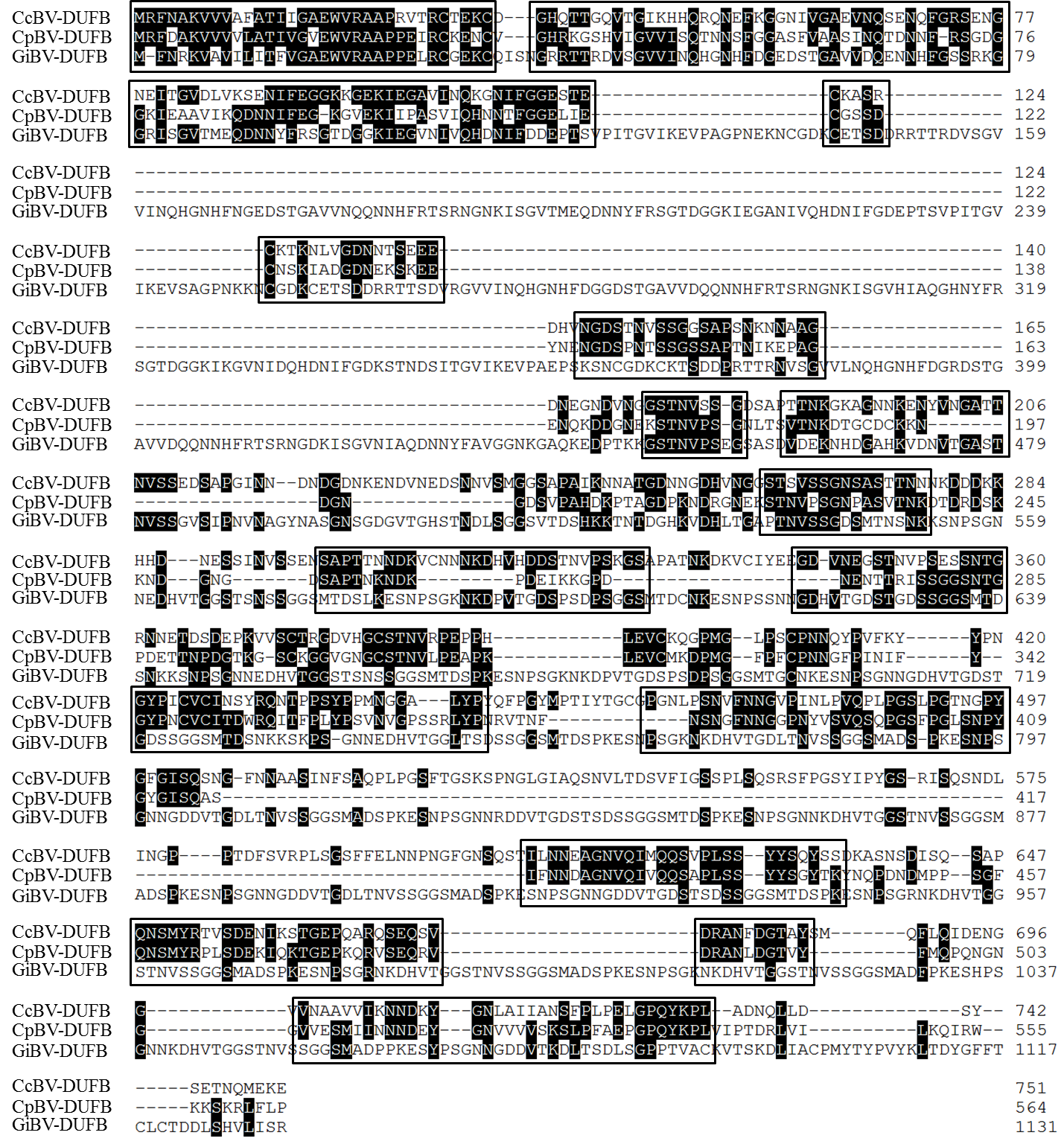

Supplement: S13 Fig — Amino acid sequences of these genes were retrieved from GenBank with accession numbers listed in S3 Table. Amino acid sequences were aligned with MEGA6 (Tamura et al., 2011). Bootstrap values on branches were obtained with 500 repetitions. The black box indicates the conserved regions among the genes. (DOC) [file pone.0200663.s013.doc]

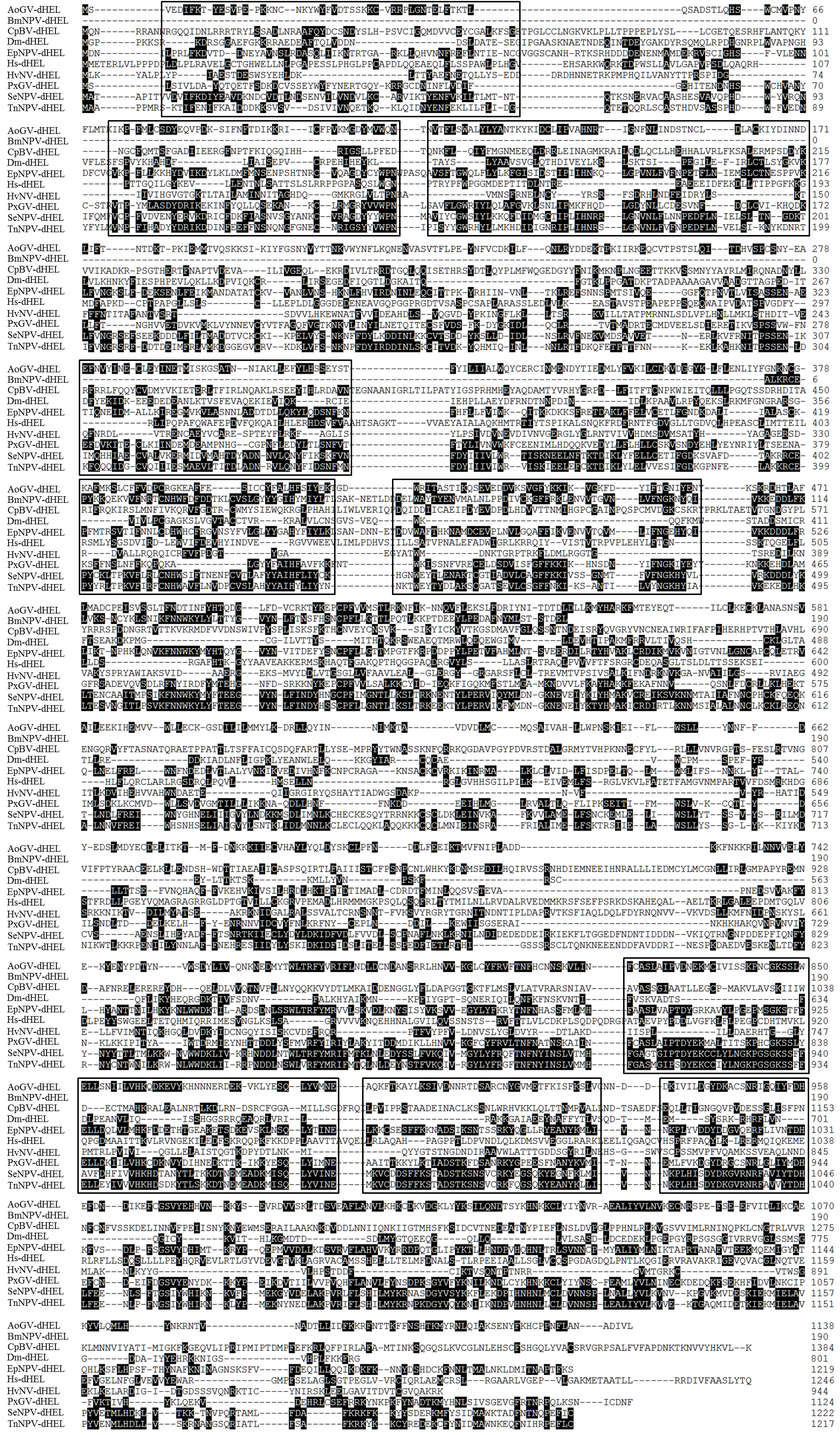

Supplement: S14 Fig — Amino acid sequences of these genes were retrieved from GenBank with accession numbers listed in S3 Table. Amino acid sequences were aligned with MEGA6 (Tamura et al., 2011). Bootstrap values on branches were obtained with 500 repetitions. The black box indicates the conserved regions among the genes. (DOC) [file pone.0200663.s014.doc]

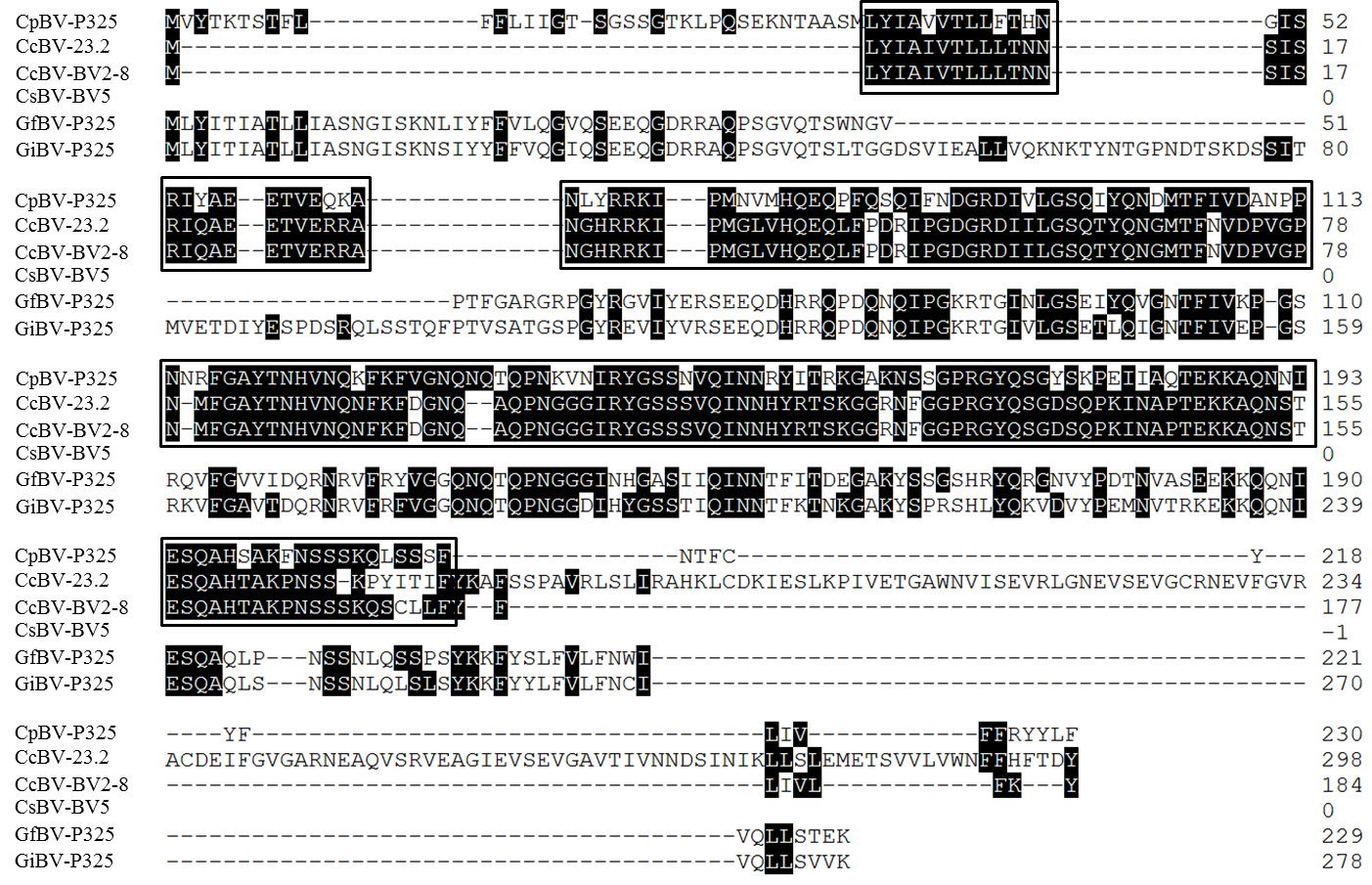

Supplement: S15 Fig — Amino acid sequences of these genes were retrieved from GenBank with accession numbers listed in S3 Table. Amino acid sequences were aligned with MEGA6 (Tamura et al., 2011). Bootstrap values on branches were obtained with 500 repetitions. The black box indicates the conserved regions among the genes. (DOC) [file pone.0200663.s015.doc]

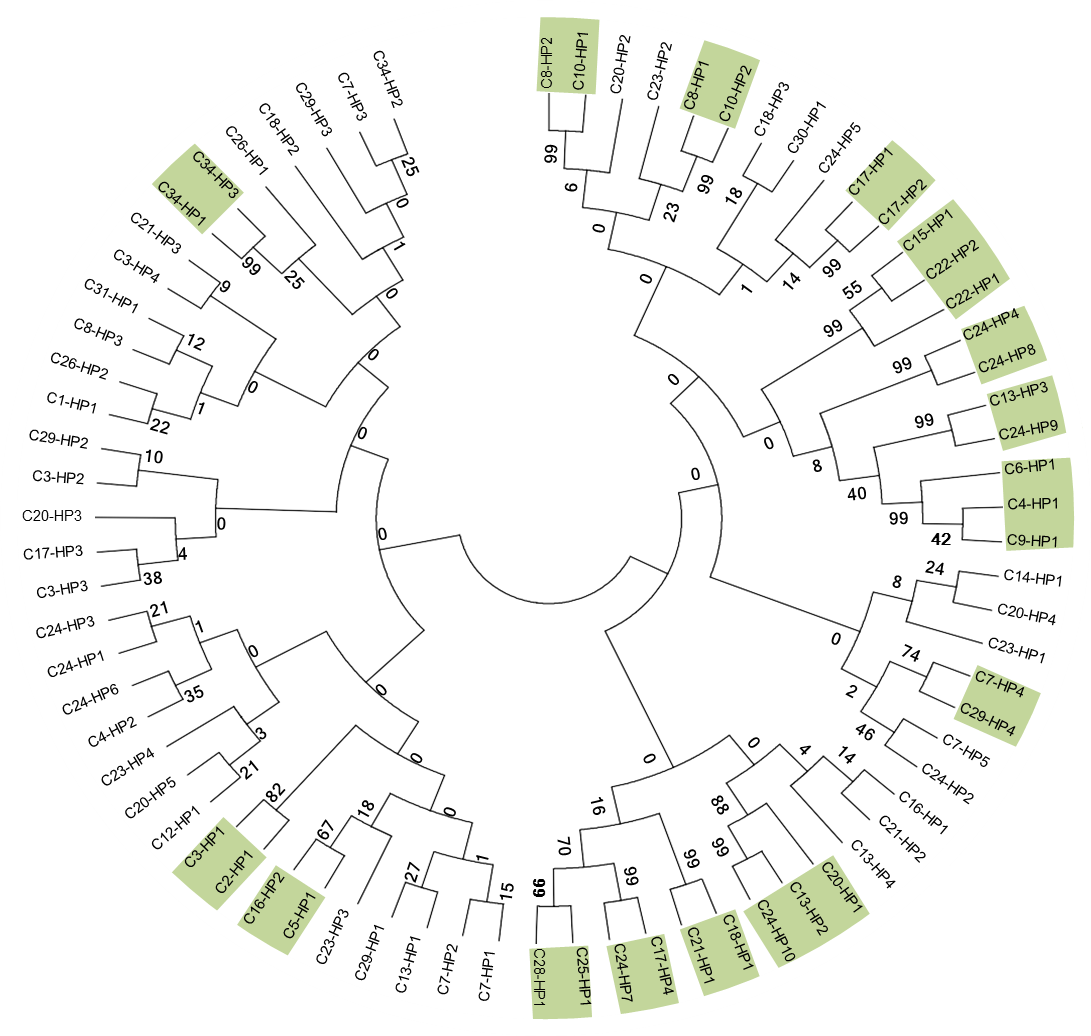

Supplement: S16 Fig — Amino acid sequences of these genes were retrieved from GenBank with accession numbers listed in S3 Table. Amino acid sequences were aligned with MEGA6 (Tamura et al., 2011). Bootstrap values on branches were obtained with 500 repetitions. (DOC) [file pone.0200663.s016.doc]
