# Supplementary material for: Persistent expression of Cotesia plutellae bracovirus genes in parasitized host, Plutella xylostella
Source: PLoS One. 2018 Jul 16;13(7):e0200663. doi: 10.1371/journal.pone.0200663 (PMC6047808; doi:10.1371/journal.pone.0200663)
Supplement: S1 Table — (DOC) [file pone.0200663.s017.doc]

**S1 Table**. RNA-Seq summary of host (*P. xylostella*) gene expression in nonparasitized (NP) and parasitized (P)

| Samples | # of trimmed reads | Reads mapping to *P. xylostella*  # of mapped reads Mapping  ratios | Reads mapping to CpBV  # of mapped reads Mapping  ratios |
| --- | --- | --- | --- |
| N1 | 145312364 | 84318467 58.0% | Not applicable |
| N5 | 148458058 | 81876641 55.2% | Not applicable |
| P1 | 118266038 | 63244339 53.5% | 6,542,881 5.5% |
| P7 | 108508176 | 28240047 26.0% | 50,479,330 46.5% |
