# Supplementary material for: Persistent expression of Cotesia plutellae bracovirus genes in parasitized host, Plutella xylostella
Source: PLoS One. 2018 Jul 16;13(7):e0200663. doi: 10.1371/journal.pone.0200663 (PMC6047808; doi:10.1371/journal.pone.0200663)
Supplement: S2 Table — (DOC) [file pone.0200663.s018.doc]

**S2 Table**. List of primers used in RT-PCR and RT-qPCR

| Genes | Direction | Sequences (5`→ 3’) |
| --- | --- | --- |
| C13-HP4 | Forward | TACCTGGCTACCGTGTACTATC |
| C13-HP4 | Reverse | TTGCGGCGAGCTTGTAAT |
| C17-HP1 | Forward | CTCACTTGAGTCGGAGGATAAC |
| C17-HP1 | Reverse | TCTCGAGAGGAGTCGATGAA |
| C17-HP3 | Forward | AATCCTCGCGAAACGGATTA |
| C17-HP3 | Reverse | GCCTGACATTCCGGTCATAATA |
| C17-HP4 | Forward | GAGTATGTTCTTATAGCACCGGATA |
| C17-HP4 | Reverse | CAGTAGGCACCAATTCACAAAC |
| C20-HP4 | Forward | CGTTGTAAAGTTCTACGGGATTTC |
| C20-HP4 | Reverse | CCAAGTGTGCCATTAGAAACAA |
| C24-HP1 | Forward | CTCTGTTACCAAGCATGGTTCT |
| C24-HP1 | Reverse | AGAAACTGGCGCCCAAC |
| C24-HP4 | Forward | ACTGGTCCTCACAGTAGTCA |
| C24-HP4 | Reverse | GAGTGGGACCACTACTACAAATC |
| C24-HP5 | Forward | CGGGTAACAACTCTCAAGACAA |
| C24-HP5 | Reverse | CTCATCATCCTCTCGCTTTCG |
| C24-HP7 | Forward | GTCGTATTGAGTTCTGCGAAAG |
| C24-HP7 | Reverse | CTCTCTTGACGAAGAGGAGAAG |
| C31-HP1 | Forward | GCCATCTGCACTTTGGATTC |
| C31-HP1 | Reverse | GACTTCGGAGCTTCAGAAAGA |
| C2-IkB7 | Forward | TGTAGCAGCCACATCATAATCC |
| C2-IkB7 | Reverse | CCCAAAGGAAGTTCGAGTTTCTA |
| C7-HP3 | Forward | TCGCAGCGTTTGAGTCTTTA |
| C7-HP3 | Reverse | TTCCCGGAATTGTGAGGATAAG |
| C7-ELP7 | Forward | CTACCAGACTACCGCACAATC |
| C7-ELP7 | Reverse | CCTCTGCTTAGAATCCGATGAC |
| C15-BEN1 | Forward | ACCTCCTGCTACATTACCTTTG |
| C15-BEN1 | Reverse | GAGGCTGATGCTCCTGAATTAT |
| C26-HP1 | Forward | TTACCGCTGCACAAGTATGG |
| C26-HP1 | Reverse | CCAACTCCGTTCGGTACAATAA |
| C26-HP2 | Forward | CTTCAGCAAATTACCGCTTGTT |
| C26-HP2 | Reverse | AGGGTATCCACTGCCTACTT |
| C26-BEN4 | Forward | CCGAGACTGCTAATCACCTTTAC |
| C26-BEN4 | Reverse | GCCCGAATCCTTGTCCATTAT |
| C32-ELP4 | Forward | GCTGCAAAGCCCTTGTTATC |
| C32-ELP4 | Reverse | CTCCCACAGCTTCATAGGTTC |
| C34-P494-1 | Forward | GATATGAAGCCCGACCCAAA |
| C34-P494-1 | Reverse | GGTGTATGCTGAGGAAGATGAG |
| C34-P494-2 | Forward | GATATGAAGCCCGACCCAAA |
| C34-P494-2 | Reverse | GGTGTATGCTGAGGAAGATGAG |
| C6-PTP26 | Forward | TGACAGCTTCCTTTCTCTCTTC |
| C6-PTP26 | Reverse | CGTGTTTGACCTCATCCATCTA |
| C8-HP1 | Forward | GCGTGTAAGCTAGATGAGGTAA |
| C8-HP1 | Reverse | AAGGATTCGGCCATGGATAG |
| C8-HP2 | Forward | TACTGCACAAACCGGATCTAAC |
| C8-HP2 | Reverse | ACCAGAGTCGGAAGGAATCT |
| C8-HP3 | Forward | TTTGCTTTGGCTCATCTTTAGC |
| C8-HP3 | Reverse | GTGACCCGAGAGGACAATAAC |
| C10-HP2 | Forward | GCGTGTAAGCTAGATGAGGTAA |
| C10-HP2 | Reverse | AAGGATTCGGCCATGGATAG |
| C17-IkB4 | Forward | CCTTGGTAGAATGGCTGTGT |
| C17-IkB4 | Reverse | CTTGCACTCTGGTTGGTAGT |
| C19-PTP15 | Forward | TCACCCGTTGCATTGTTAGTA |
| C19-PTP15 | Reverse | GGCGCAGAAGAGAAGATTCA |
| C19-PTP16 | Forward | CTGGTACATCGTCTTCCTCAAA |
| C19-PTP16 | Reverse | TCGGTACTTTCTACGCTTGTC |
| C35-dHEL | Forward | GCGGTTTCGTCGATTGTTTC |
| C35-dHEL | Reverse | GTGCTGTGATGTCGTGTCTAT |
| C33-E94K5 | Forward | GTACAAAGCCGGAGAGCATTA |
| C33-E94K5 | Reverse | GCTTGCCATTTCGATACATCAC |
| RL32 | Forward | ATGCCCAACATTGGTTACGG |
| RL32 | Reverse | TTCGTTCTCCTGGCTGCGGA |
