# Supplementary material for: Persistent expression of Cotesia plutellae bracovirus genes in parasitized host, Plutella xylostella
Source: PLoS One. 2018 Jul 16;13(7):e0200663. doi: 10.1371/journal.pone.0200663 (PMC6047808; doi:10.1371/journal.pone.0200663)
Supplement: S3 Table — (DOC) [file pone.0200663.s019.doc]

**S3 Table**. List of NCBI-GenBank accession numbers used in phylogenetic analyses

| Group | Accession No. | Group | Accession No. | Group | Accession No. |
| --- | --- | --- | --- | --- | --- |
| CpBV_BEN1 | AEE09526.1 | CpBV_IkB-1 | AEE09521.1 | CpBV_ELP1 | AEE09500.1 |
| CpBV_BEN2 | AEE09452.1 | CpBV_IkB-2 | AEE09523.1 | CpBV_ELP2 | AEE09505.1 |
| CpBV_BEN3 | AEE09584.1 | CpBV_IkB-3 | AEE09535.1 | CpBV_ELP3 | AEE09594.1 |
| CpBV_BEN4 | AEE09580.1 | CpBV_IkB-4 | AEE09536.1 | CpBV_ELP4 | AEE09600.1 |
| CpBV_BEN5 | AEE09528.1 | CpBV_IkB-5 | AEE09578.1 | CpBV_ELP5 | AEE09456.1 |
| CpBV_BEN6 | AEE09539.1 | CpBV_IkB-6 | AEE09586.1 | CpBV_ELP6 | AEE09469.1 |
| CpBV_BEN7 | AEE09512.1 | CpBV_IkB-7 | AEE09454.1 | CpBV_ELP7 | AEE09482.1 |
| CpBV_BEN8 | AEE09493.1 | CcBV_IkB-1 | CAE47492.1 | CcBV_ELP1 | YP_184799.1 |
| CpBV_BEN9 | AEE09484.1 | CcBV_IkB-2 | CAE47493.1 | CcBV_ELP2 | YP_184760.1 |
| CpBV_BEN10 | AEE09554.1 | CcBV_IkB-3 | CAE47494.1 | CcBV_ELP3 | YP_184761.1 |
| CpBV_BEN11 | AEE09581.1 | CcBV_IkB-4 | CCQ71346.1 | CcBV_ELP4 | YP184789 |
| CpBV_BEN12 | AEE09583.1 | CcBV_IkB-5 | YP_184870.1 | CcBV_ELP5 | CAG17419.1 |
| CcBV_BEN-2 | CCQ71194.1 | CcBV_IkB-6 | CCQ71374.1 | CcBV_ELP6 | CCQ71203.1 |
| CcBV_BEN-3 | CCQ71096.1 | CcBV_IkB-7 | CCB96378.1 | CpBV-CTL1 | AEE09562.1 |
| CcBV_BEN-4 | CCQ71118.1 | CcBV_IkB-8 | CCB96379.1 | CpBV-CTL2 | AEE09593.1 |
| CcBV_BEN-5 | CCQ71332.1 | CcBV_IkB-9 | CCQ71367.1 | CcBV-CTL | CCQ71085.1 |
| CcBV_BEN-7 | CCQ71277.1 | GiBV_IkB | ACE75325.1 | CrBV-CTL | AAO74641.1 |
| CcBV_BEN-8 | CCQ71117.1 | GfBV_IkB | ACE75201.1 | GfBV-CTL | ACE75063.1 |
| CcBV_BEN-9 | CCQ71095.1 | TnBV_IkB | CAE47441.1 | GiBV-CTL | ABK56997.1 |
| CcBV_BEN-10 | CCQ71084.1 | CpBV-E94K1 | AEE09499.1 | CpBV-P494_1 | AEE09605.1 |
| CcBV_BEN-11 | CCQ71144.1 | CpBV-E94K2 | AEE09498.1 | CpBV-P494_2 | AEE09606.1 |
| CcBV_BEN-12 | CCB96390.1 | CpBV-E94K3 | AEE09497.1 | CcBV-P494 | CCQ71139.1 |
| CcBV_BEN-14 | CCB96386.1 | CpBV-E94K4 | AEE09494.1 | GiBV-P494 | AAP87442.1 |
| MdBV_BEN | Q5MAE6.1 | CpBV-E94K5 | AEE09601.1 | GfBV-P494 | ACE75089.1 |
| MdBV_BEN | XP_014299756.1 | CcBV-E94K1 | CCQ71306.1 | CpBV-CrV1 | AEE09597.1 |
| MdBV_BEN | XP_014294990.1 | CcBV-E94K2 | CCQ71305.1 | CsBV-CrV1 | AGO14478.1 |
| CpBV_SRP1 | AEE09516.1 | CpBV-CRP1 | AEE09513.1 | CcBV-CrV1 | AAL82896.1 |
| CpBV_SRP2 | AEE09545.1 | CpBV-CRP2 | AEE09573.1 | CrBV-CrV1 | AAB40714.1 |
| CpBV_SRP3 | AEE09547.1 | CpBV-CRP3 | AEE09459.1 | CfBV-CrV1 | ABC96861.1 |
| CpBV_SRP4 | AEE09551.1 | CcBV-CRP1 | YP_184840.1 | GiBV-CrV1 | ABK57000.1 |
| CpBV_SRP5 | AEE09564.1 | CcBV-CRP2 | YP_184841.1 | CpBV-DUFB | AEE09468.1 |
| CpBV_SRP6 | AEE09567.1 | CcBV-CRP3 | CCQ71239.1 | CcBV-DUFB | YP_184785.1 |
| CcBV_SRP1 | CCQ71172.1 | CcBV-CRP4 | CCQ71266.1 | GiBV-DUFB | ABK57053.1 |
| CcBV_SRP2 | CCQ71176.1 | CpBV-CST1 | AEE09558.1 | CpBV-vH4 | AEE09495.1 |
| CcBV_SRP3 | CCQ71184.1 | CrBV-CST | ACD50833.1 | CcBV-vH4 | CAG17417.1 |
| CcBV_SRP4 | CCQ71205.1 | CsBV-CST | ACD50824.1 | CgBV-vH4 | ABH10013.1 |
| CcBV_SRP5 | CCQ71216.1 | CfBV-CST | ACD50812.1 | CsBV-vH4 | ALF62335.1 |
| CcBV_SRP6 | CCQ71236.1 | CmBV-CST | ACD50819.1 | AoGBV-dHEL | NP_872530.1 |
| CcBV_SRP7 | CCQ71252.1 | CchBV-CST | ACD50806.1 | Bm-dHEL | AAB28154.1 |
| CcBV_SRP8 | CCQ71271.1 | CcBV-CST1 | YP_184846.1 | CpBV-dHEL | AEE09607.1 |
| CpBV-P325 | AEE09529.1 | CcBV-CST2 | YP_184849.1 | Dm-HEL | AAA74931.1 |
| CcBV-P325 | YP_184860.1 | CcBV-CST3 | YP_184851.1 | EpNPV-dHEL | NP_203252.1 |
| CcBV-P325 | CCQ71092.1 | CpBV-PTP20 | AEE09590.1 | Hs-dHEL | AAB67978.1 |
| CskBV-P325 | CCQ19206.1 | CpBV-PTP21 | AEE09467.1 | HvNPV-dHEL | AFV50267.1 |
| GfBV-P325 | ACE75118.1 | CpBV-PTP22 | AEE09466.1 | PxGV-dHEL | AMQ35682.1 |
| GiBV-P325 | ABK57035.1 | CpBV-PTP23 | AEE09465.1 | TnNPV-dHEL | YP_308971.1 |
| CpBV-PTP1 | AEE09511.1 | CpBV-PTP24 | AEE09463.1 | SeNPV-dHEL | DG72974.1 |
| CpBV-PTP2 | AEE09510.1 | CpBV-PTP25 | AEE09462.1 | CcBV-PTPL | YP_184763.1 |
| CpBV-PTP3 | AEE09509.1 | CpBV-PTP26 | AEE09472.1 | CcBV-PTPM | YP_184764.1 |
| CpBV-PTP4 | AEE09508.1 | CpBV-PTP27 | AEE09473.1 | CcBV-PTPN | YP_184809.1 |
| CpBV-PTP5 | AEE09507.1 | CpBV-PTP28 | AEE09474.1 | CcBV-PTPO | YP_184783.1 |
| CpBV-PTP6 | AEE09506.1 | CpBV-PTP29 | AEE09475.1 | CcBV-PTPP | YP_184765.1 |
| CpBV-PTP7 | AEE09496.1 | CpBV-PTP30 | AEE09476.1 | CcBV-PTPQ | YP_184766.1 |
| CpBV-PTP8 | AEE09503.1 | CpBV-PTP31 | AEE09477.1 | CcBV-PTPR | CAG17418.1 |
| CpBV-PTP9 | AEE09502.1 | CpBV-PTP32 | AEE09489.1 | CcBV-PTPS | YP_184805.1 |
| CpBV-PTP10 | AEE09504.1 | CpBV-PTP33 | AEE09490.1 | CcBV-PTPT | CAG17428.1 |
| CpBV-PTP11 | AEE09519.1 | CcBV-PTPA | CCQ71368.1 | CcBV-PTPU | YP_184822.1 |
| CpBV-PTP12 | AEE09520.1 | CcBV-PTPalpha | CCQ71291.1 | CcBV-PTPV | CCQ71345.1 |
| CpBV-PTP13 | AEE09524.1 | CcBV-PTPB | YP_184757.1 | CcBV-PTPW | YP_184827.1 |
| CpBV-PTP14 | AEE09525.1 | CcBV-PTPC | YP_184807.1 | CcBV-PTPX | CCQ71295.1 |
| CpBV-PTP15 | AEE09542.1 | CcBV-PTPD | YP_184767.1 | CcBV-PTPY | CCQ71294.1 |
| CpBV-PTP16 | AEE09543.1 | CcBV-PTPE | YP_184804.1 | CcBV-PTPZ | CCQ71293.1 |
| CpBV-PTP17 | AEE09585.1 | CcBV-PTPH | YP_184782.1 | MdBV-PTP | Q5I146.1 |
| CpBV-PTP18 | AEE09588.1 | CcBV-PTPI | YP_184758.1 | TnBV-PTP | CAG25452.1 |
| CpBV-PTP19 | AEE09589.1 | CcBV-PTPK | YP_184762.1 | CpBV-C8-HP3 | AEE09487.1 |
| CpBV-C1HP1 | AEE09451.1 | CpBV-C28-HP1 | AEE09587.1 | CpBV-C13-HP3 | AEE09517.1 |
| CpBV-C2-HP1 | AEE09453.1 | CpBV-C29-HP1 | AEE09591.1 | CpBV-C17-HP3 | AEE09534.1 |
| CpBV-C3-HP1 | AEE09455.1 | CpBV-C30-HP1 | AEE09598.1 | CpBV-C18-HP3 | AEE09541.1 |
| CpBV-C4-HP1 | AEE09461.1 | CpBV-C31-HP1 | AEE09599.1 | CpBV-C20-HP3 | AEE09548.1 |
| CpBV-C5-HP1 | AEE09470.1 | CpBV-C34-HP1 | AEE09602.1 | CpBV-C21-HP3 | AEE09555.1 |
| CpBV-C6-HP1 | AEE09471.1 | CpBV-C3-HP2 | AEE09458.1 | CpBV-C23-HP3 | AEE09561.1 |
| CpBV-C7-HP1 | AEE09478.1 | CpBV-C4-HP2 | AEE09464.1 | CpBV-C24-HP3 | AEE09568.1 |
| CpBV-C8-HP1 | AEE09486.1 | CpBV-C7-HP2 | AEE09479.1 | CpBV-C34-HP3 | AEE09604.1 |
| CpBV-C9-HP1 | AEE09488.1 | CpBV-C8-HP2 | AEE09485.1 | CpBV-C29-HP3 | AEE09595.1 |
| CpBV-C10-HP1 | AEE09492.1 | CpBV-C10-HP2 | AEE09491.1 | CpBV-C3-HP4 | AEE09460.1 |
| CpBV-C12-HP1 | AEE09501.1 | CpBV-C13-HP2 | AEE09515.1 | CpBV-C7-HP4 | AEE09480.1 |
| CpBV-C13-HP1 | AEE09514.1 | CpBV-C16-HP2 | AEE09531.1 | CpBV-C13-HP4 | AEE09518.1 |
| CpBV-C14-HP1 | AEE09522.1 | CpBV-C17-HP2 | AEE09533.1 | CpBV-C17-HP4 | AEE09537.1 |
| CpBV-C15-HP1 | AEE09527.1 | CpBV-C18-HP2 | AEE09540.1 | CpBV-C20-HP4 | AEE09549.1 |
| CpBV-C16-HP1 | AEE09530.1 | CpBV-C20-HP2 | AEE09546.1 | CpBV-C23-HP4 | AEE09563.1 |
| CpBV-C17-HP1 | AEE09532.1 | CpBV-C21-HP2 | AEE09553.1 | CpBV-C24-HP4 | AEE09569.1 |
| CpBV-C18-HP1 | AEE09538.1 | CpBV-C22-HP2 | AEE09557.1 | CpBV-C29-HP4 | AEE09596.1 |
| CpBV-C20-HP1 | AEE09544.1 | CpBV-C23-HP2 | AEE09560.1 | CpBV-C7-HP5 | AEE09481.1 |
| CpBV-C21-HP1 | AEE09552.1 | CpBV-C24-HP2 | AEE09566.1 | CpBV-C20-HP5 | AEE09550.1 |
| CpBV-C22-HP1 | AEE09556.1 | CpBV-C26-HP2 | AEE09579.1 | CpBV-C24-HP5 | AEE09570.1 |
| CpBV-C23-HP1 | AEE09559.1 | CpBV-C29-HP2 | AEE09592.1 | CpBV-C24-HP6 | AEE09571.1 |
| CpBV-C24-HP1 | AEE09565.1 | CpBV-C34-HP2 | AEE09603.1 | CpBV-C24-HP7 | AEE09572.1 |
| CpBV-C25-HP1 | AEE09577.1 | CpBV-C3-HP3 | AEE09457.1 | CpBV-C24-HP8 | AEE09574.1 |
| CpBV-C26-HP1 | AEE09582.1 | CpBV-C7-HP3 | AEE09483.1 | CpBV-C24-HP9 | AEE09487.1 |
| CpBV-C24-HP9 | AEE09576.1 | CpBV-C24-HP10 | AEE09575.1 |  |  |
